# Life Within a Contaminated Niche: Comparative Genomic Analyses of an Integrative Conjugative Element ICE*nah*CSV86 and Two Genomic Islands From *Pseudomonas bharatica* CSV86^T^ Suggest Probable Role in Colonization and Adaptation

**DOI:** 10.3389/fmicb.2022.928848

**Published:** 2022-07-06

**Authors:** Balaram Mohapatra, Harshit Malhotra, Prashant S. Phale

**Affiliations:** Department of Biosciences and Bioengineering, Indian Institute of Technology Bombay, Mumbai, India

**Keywords:** *Pseudomonas*, integrative conjugative element (ICE), comparative and functional genomics, phylogenomics, naphthalene metabolism, Co-Zn-Cd tolerance, niche adaptation, evolution-speciation

## Abstract

Comparative genomic and functional analyses revealed the presence of three genomic islands (GIs, >50 Kb size): ICE*nah*CSV86, *Pseudomonas bharatica* genomic island-1 (PBGI-1), and PBGI-2 in the preferentially aromatic-degrading soil bacterium, *Pseudomonas bharatica* CSV86^T^. Site-specific genomic integration at or near specific transfer RNAs (tRNAs), near-syntenic structural modules, and phylogenetic relatedness indicated their evolutionary lineage to the type-4 secretion system (T4SS) ICE*clc* family, thus predicting these elements to be integrative conjugative elements (ICEs). These GIs were found to be present as a single copy in the genome and the encoded phenotypic traits were found to be stable, even in the absence of selection pressure. ICE*nah*CSV86 harbors naphthalene catabolic (*nah*-*sal*) cluster, while PBGI-1 harbors Co-Zn-Cd (*czc*) efflux genes as cargo modules, whereas PBGI-2 was attributed to as a mixed-function element. The ICE*nah*CSV86 has been reported to be conjugatively transferred (frequency of 7 × 10^–8^/donor cell) to *Stenotrophomonas maltophilia* CSV89. Genome-wide comparative analyses of aromatic-degrading bacteria revealed *nah*-*sal* clusters from several *Pseudomonas* spp. as part of probable ICEs, syntenic to conjugatively transferable ICE*nah*CSV86 of strain CSV86^T^, suggesting it to be a prototypical element for naphthalene degradation. It was observed that the plasmids harboring *nah*-*sal* clusters were phylogenetically incongruent with predicted ICEs, suggesting genetic divergence of naphthalene metabolic clusters in the *Pseudomonas* population. Gene synteny, divergence estimates, and codon-based *Z*-test indicated that ICE*nah*CSV86 is probably derived from PBGI-2, while multiple recombination events masked the ancestral lineage of PBGI-1. Diversifying selection pressure (dN-dS = 2.27–4.31) imposed by aromatics and heavy metals implied the modular exchange-fusion of various cargo clusters through events like recombination, rearrangement, domain reshuffling, and active site optimization, thus allowing the strain to evolve, adapt, and maximize the metabolic efficiency in a contaminated niche. The promoters (P*nah* and P*sal*) of naphthalene cargo modules (*nah*, *sal*) on ICE*nah*CSV86 were proved to be efficient for heterologous protein expression in *Escherichia coli*. GI-based genomic plasticity expands the metabolic spectrum and versatility of CSV86^T^, rendering efficient adaptation to the contaminated niche. Such isolate(s) are of utmost importance for their application in bioremediation and are the probable ideal host(s) for metabolic engineering.

## Introduction

Horizontal transfer of gene(s) “*en masse*” through mobile genetic elements [MGEs: genomic islands (GIs)/integrative conjugative elements (ICEs), prophages, plasmids, etc.] encoding adaptive or niche colonization traits is the decisive factor for species diversification, evolution, and pan-genomic stability in prokaryotes (Burrus et al., 2002; Rodríguez-Beltrán et al., 2020). Amongst MGEs, GIs are strain-specific segments of the bacterial genome, which are often found to be present at limited chromosomal locations. Depending on the functions they encode and the specific lifestyle of the bacterium, GIs can be classified into metabolic, pathogenicity, symbiosis, fitness, or resistance islands (Dobrindt et al., 2004; Juhas et al., 2009). GIs located on chromosomes are typically flanked by direct repeats and are inserted at the 3′ end of a transfer RNA (tRNA) gene. Further, these elements harbor transposase(s) or integrase(s) that are required for chromosomal integration and excision along with other mobile genes, in a strain or clone-specific manner. GIs are usually differentiated from the rest of the core genome by their atypical G + C contents and oligonucleotide compositions, with steep gradients at their boundaries (Reva and Tümmler, 2005). Comparative genetic mapping and evolutionary analyses have uncovered diverse GIs in bacterial taxa, particularly amongst proteobacterial members, with pathogenicity islands of *Enterobacteriaceae* being the most studied GIs (Hacker and Carniel, 2001).

Genomic islands and ICEs have structural similarities, such as the presence of integrase/excisionase or conserved core gene regions that belong to a common group of MGEs, often termed as ICElands (van der Meer and Sentchilo, 2003). While GIs might be self-transmissible and/or unstable, ICEs are distinguished by their conjugative, integrative, and stable/consistent behavior (Wozniak and Waldor, 2010; Delavat et al., 2017). Classically, the core module(s) of ICEs comprise (i) an integrase (*xer*C, responsible for ICE excision to form circular intermediate), often assisted by an excisionase or recombination directionality factors (RDFs), (ii) type-4 secretion system (T4SS) (for conjugative transfer), and (iii) partition (*par*)- maintenance cluster, ensuring successful horizontal transmission amongst near-relatives (Burrus, 2017; Liu et al., 2019). Most importantly, the “cargo gene array(s)” which differ from ICE to ICE, encode diverse metabolic traits which provide survival fitness to the host in a particular niche (Wozniak and Waldor, 2010). Although both GIs and ICEs are synonymous, there are no clear demarcations on their evolution and/or mode of mobilization.

Genomic islands or ICEs harboring genetic modules for the degradation of aromatics and tolerance/resistance to heavy metals (as functional cargo) are of immense scientific interest due to their potential application in bioremediation and metal detoxification/recovery. With large-scale discharge, resonance-stabilized structures, hydrophobicity, and persistent nature of aromatics and toxicity of metals, selection pressure is exerted on the microbial communities, leading to the evolution of efficient metabolic pathways or tolerance mechanisms (Nojiri et al., 2009; Fuentes et al., 2014; Phale et al., 2019). This requires a high degree of genetic exchange and recombination events within the community mediated by mobile genetic elements through horizontal gene transfer (HGT) process. Till date, few GIs and ICEs, such as *phn* island (phenanthrene metabolism), *clc* GI (later classified as ICE*clc*B13; chlorocatechol metabolism), ICE*clc*LB400 (chlorinated biphenyl, chlorocatechol), ICE*clc*JB2 (*o*-halobenzoates and *o*-hydroxybenzoate), ICE*XTD* (xylene-toluene), ICE*bph-sal* (biphenyl-salicylate), and ICE*Tn4371* family (Co, Zn, Cd, Cr, As, and Hg resistance) have been characterized (ICEBerg 2.0 database^1^; Liu et al., 2019). ICE*clc*B13 (105 Kb) of *Pseudomonas knackmussii* B13 encoding *ortho*-cleavage of chlorocatechol (*clc*) and aminophenol (*amp*) is a functionally characterized prototypical element (Gaillard et al., 2006; Miyazaki et al., 2015). However, the coexistence of multiple GIs (either of similar or hybrid nature) encoding degradation/tolerance traits in a single bacterium has been rarely observed (Badhai and Das, 2016; Liu et al., 2019).

An Indian isolate, strain CSV86^T^ (formerly *Pseudomonas putida*, recently effectively described as *Pseudomonas bharatica*), isolated from petrol-station soil contaminated with petroleum products preferentially metabolizes various aromatic compounds over glucose and displays the following carbon source utilization hierarchy: aromatics ≥ organic acid > glucose (Basu et al., 2006; Phale et al., 2021; Mohapatra et al., 2022). This unique preferential naphthalene degradation trait is stable, chromosomally encoded, and was found to be transferred to *Stenotrophomonas maltophilia* CSV89 through conjugation at a very low frequency (7 × 10^–8^/donor cell) (Basu and Phale, 2008). Besides, strain CSV86^T^ is able to tolerate up to 2 mM concentration of heavy metals (Cd, Zn, Co, Cu, and As) during growth on aromatics (Paliwal et al., 2014). In addition to these unique traits, the absence of plasmid and other pathogenicity and virulence factors renders strain CSV86^T^ an ideal candidate for pollutant bioremediation and host/*chassis* for metabolic engineering applications (Phale et al., 2021).

Initial genomic analyses of strain CSV86^T^ revealed the presence of a large extent of MGEs (GIs, prophages, probable ICEs, and insertion sequences), amounting to ∼19% of its total genomic content (6.79 Mb), responsible for the genomic plasticity of this isolate (Mohapatra et al., 2022). To delineate the extent, functionality, and evolutionary significance of MGEs in strain CSV86^T^, combined comparative- and functional-genome minings and molecular analyses were performed. Analyses revealed the role and significance of MGEs in adaptation, niche colonization, and species competitive behavior of strain CSV86^T^ at the contaminated site.

## Materials and Methods

### Growth and Culture Conditions of *Pseudomonas bharatica* CSV86^T^

*Pseudomonas bharatica* CSV86^T^ (= MTCC 2445^T^ = ICMP 13424^T^ = JCM 34937^T^) which metabolizes benzoate, naphthalene, and phenylpropanoids, amongst others was used in this study (Mahajan et al., 1994; Mohapatra et al., 2022). The strain was routinely grown on a minimal salt medium (MSM, 150 mL, pH 7.5; Basu et al., 2003) supplemented aseptically with naphthalene or benzoate (0.1%, w/v) or glucose (0.25%, v/v) as the sole source of carbon and energy at 30°C for 12 h at 200 rpm.

### Identification and Characterization of Genomic Islands From *Pseudomonas bharatica* CSV86^T^

The whole-genome shotgun sequencing project of strain CSV86^T^ was previously deposited at DDBJ/EMBL/GenBank under the accession number AMWJ00000000 with the first/standard version as AMWJ01000000 (Paliwal et al., 2014). The hybrid PacBio-GS FLX hybrid assembly based genome sequencing project has been deposited as the updated advanced version: AMWJ02000000 with BioProject: PRJNA65243 and BioSample: SAMN02472262 (Mohapatra et al., 2022). The GI replicons (corresponding nucleotide positions) are represented in the genome AMWJ02000000.

To identify the regions of genomic plasticity (RGPs) in strain CSV86^T^, advanced version of the genomic draft (AMWJ02000000) of CSV86^T^ was analyzed by Microscope-Genoscope (MaGe^2^) and IslandViewer 4.0^3^ using IslandPick, IslandPath-DIMOB, SIGI-HMM, and Islander prediction methods. The RGPs were manually curated to filter biases (false-positive and negative) using the following criteria: (i) presence of mobility genes (integrases/recombinases, and transposases); (ii) structural RNAs (tRNAs); (iii) atypical genomic content (GC mol% content, GC skewness, and codon usage); and (iv) presence of phenotypic trait cassettes (antibiotic/toxic compounds resistance, virulence, or substrate metabolism). To categorize RGPs, different methods were employed: (i) BLASTn searches against the ICEBerg database 2.0^4^ and PHASTER^5^ with the *E*-value ≤ 10^–5^ and sequence coverage ≥50%; (ii) homology search against nr- and RefSeq-database; (iii) evaluation of t-RNAs (tRNA Scan-SE) followed by site-specific integrase/relaxase; and (iv) presence of mobile element and hypothetical proteins. Besides, repeats (direct/inverted) were identified to define the boundaries of these elements. RGPs were identified as GIs and suspected to be ICEs, thus annotated using the RAST-tk pipeline and compared with the reported reference ICEs in ICEBerg 2.0 database (Table 1). Genes located on these suspected ICEs were categorized into orthologous groups using OrthoVenn2^6^ with an *E*-value cut-off of 10^–5^. Localized reciprocal best-hit of ICE genes was performed using the orthoANI tool (OAT) of the Ez-BioCloud server.^7^ Other genomic features (gene synteny, GC skewness, GC content mol%, and pairwise comparisons) were analyzed using Artemis Comparison Tool (ACT, release 18.1.0) and Mauve progressive alignment in MAUVE [version 20150226 build 10 (c)]. To confirm the integrative nature of the predicted ICEs, the origin of replication/transfer (*ori*C/T) was identified using both the DoriC database^8^ and manual curation using *Pseudomonas* spp. and *Escherichia coli* consensus sequences. To validate the genomic prediction and appropriate organization of the suspected ICEs, genome drafts of strain CSV86^T^ (AMWJ01 and AMWJ02) were aligned, gaps were amplified using custom-designed primers (Supplementary Table 1 and Supplementary Figure 1), and sequenced. For details of the methodology, refer to “Supplementary Material.”

**TABLE 1:** Comparative analysis of three GIs, suspected to be ICEs, from *Pseudomonas bharatica* CSV86^T^ and syntenic members of ICE*clc* family used in this study.

ICE name	Organism harboring	Genome accession*	Size (Kb)	GC mol%	Δ GC%	Function encoding	References
ICE*nah*CSV86	*Pseudomonas bharatica* CSV86^T^	AMWJ02.1	105	62.5	0.7	Naphthalene degradation	This study
PBGI-1	*Pseudomonas bharatica* CSV86^T^	AMWJ02.2	89.8	53.9	9.3	Heavy metal (Co, Zn, Cd) resistance	This study
PBGI-2	*Pseudomonas bharatica* CSV86^T^	AMWJ02.2	91.9	56.07	7.13	Mixed function	This study
ICE*clc*B13	*Pseudomonas knackmussii* B13	HG322950	102.7	62.51	3.49	3-Chloro benzoate	Sentchilo et al., 2009
ICE*clc*JB2	*Pseudomonas aeruginosa* JB2	CP028917.1	123.2	61.5	3.5	*o*-Chloro benzoate degradation	Obi et al., 2018
ICE*Pae*LES	*Pseudomonas aeruginosa* LESB58	NC_011770	111.1	64.42	2.1	Mercuric resistance	Liu et al., 2019
PAGI-2	*Pseudomonas aeruginosa* C	NC_009539	104.9	64.71	1.8	Heavy metal (Hg) resistance, cytochrome biogenesis	Klockgether et al., 2007
ICE*Pae*690	*Pseudomonas aeruginosa* FFUP_PS_690	AP014646	86.2	63.05	3.05	Carbapenem resistance, virulence	Botelho et al., 2018
ICE*Xca*Ve85-1	*Xanthomonas campestris* 85-10	NC_007508	109.2	64.06	0.7	Streptomycin resistance	Liu et al., 2019
ICE*Xca*Ca8004-1	*Xanthomonas campestris* 8004	NC_007086	98.9	60.48	4.48	Unknown	Qian et al., 2005
ICE*Bxe*LB400-1	*Burkholderia xenovorans* LB400	NC_007951	122.8	61.71	1.05	Drug resistance	Chain et al., 2006
ICE*Cme*CH34-1	*Cupriavidus metallidurans* CH34	NC_007973	109.5	64.67	−0.85	Mercuric resistance	Janssen et al., 2010
ICE*Hae*ULPAs-1	*Herminiimonas arsenicoxydans*	NC_009138	88.1	62.85	−8.56	Arsenic resistance	Liu et al., 2019
ICE*AciJ*S42-1	*Acidovorax* sp. JS42	NC_008782	119.9	65.22	0.83	Unknown	Ryan et al., 2009
ICE*XTD*	*Azoarcus* sp. CIB	NC_006513	173.7	59	6.5	Xylene, toluene degradation	Zamarro et al., 2016
ICE*Axy*A8-1	*Achromobacter xylosoxidans* A8	NC_014640	83.1	64.78	1.21	Heavy metals (Mercuric, Copper, Arsenic)	Strnad et al., 2011
ICE-GI1	*Bordetella petrii* DSM 12804	NC_010170	255.5	61.73	3.75	Heavy metals, antibiotic resistance/efflux	Lechner et al., 2009

*These are predicted as ICEs in the current study and are reported to be the part of the genomes of corresponding organisms.

### Phylogenomic Analyses and Diversifying Selection of Core and Cargo Modules

The three suspected ICEs and their similar/syntenic homologs were subjected to multiple alignment using CLUSTALW (without gaps and including all positions) in MEGA 7.0 (Kumar et al., 2016). Phylogenetic reconstruction was performed using the neighbor-joining (NJ) method based on 1,000 bootstrap iterations using Jukes–Cantor distance model, where the robustness of the trees was assessed using maximum-likelihood (ML) and minimum-evolution (ME) algorithms. Owing to high synteny and conservedness of core genes within ICE classes, amino acid sequences were subjected to phylogenetic reconstruction. Multiple alignment of concatenated amino acid sequences of T4SS-T4CP was performed using MUSCLE, and phylogeny was deciphered through the ML method involving Jones–Thornton–Taylor (JTT) substitution matrix with 1,000 bootstrap revaluation in MEGA 7.0. Additionally, individual ICE core proteins like integrase, transposase as well as cargo proteins (*nah*-*sal* cluster, NahR regulator, and *czc* heavy metal tolerance cluster) were used for phylogenetic interpretations using Poisson’s correction and Kimura-2-parameter models involving ML algorithm in MEGA 7.0. The robustness and reliability of phylogenetic trees were evaluated by 1,000 bootstrap replications. Further, overall ICE-related indices (average nucleotide-/amino acid identity, pattern disparity index, and substitution estimation) were calculated using the OrthoANI (OAT) tool of the EzBiocloud server,^9^ GGDC tool of Type Strain Genome Server (TYGS^10^) with recommended settings, and MEGA 7.0, respectively. Codon-based *Z*-Test of selection (diversifying/purifying-/neutral- selection) was performed by codon-by-codon alignment in MEGA 7.0 followed by dN-dS and dN/dS vs. dS computation to obtain the mean divergence rate. To validate the genomic divergence, the total GC mol% of the genomes and the mean deviation (ΔG + C mol%) were calculated using the G + C calculator. Nucleotide sequences of OriC and associated elements (*par*B, *par*A, *gid*B, *yid*C, *rnp*A, *dna*A, *dna*N, *rec*F, and *gyr*B) were concatenated, aligned using CLUSTALW, and phylogeny was deciphered in MEGA 7.0.

### Stability of ICE*nah*CSV86 and *Pseudomonas bharatica* Genomic Island-1 in Strain CSV86^T^

The genetic and phenotypic stability of these elements in strain CSV86^T^ was evaluated by performing growth experiments in the absence of selection pressure for 60 generations [corresponding to ∼6 generations in a single growth cycle in batch culture (t_d_ = 1.11 h), thus a total of 10 batch transfers]. Strain CSV86^T^ was grown on MSM (150 mL, pH 7.5) amended with benzoate (0.1%, w/v) as the sole source of carbon and energy. Culture grown on benzoate (growth OD_540 *nm*_ = 1.5–1.7) for 0th (control condition), 30th (5 batch transfers), and 60th (10 batch transfers) generations was transferred to MSM (150 mL, pH 7.5) containing: (i) naphthalene (0.1%, w/v) as the sole source of carbon, and (ii) benzoate plus heavy metals (CZC: Co^2+^, Zn^2+^, and Cd^2+^, each at 1 mM). Culture growth was assessed by measuring the optical density (OD_540 *nm*_) at periodic intervals for naphthalene-amended conditions. While for heavy metals, growth was monitored at 12 and 24 h intervals. Minimum inhibitory concentration (MIC) was determined by tube dilution method, where cells of strain CSV86^T^ were inoculated into Luria broth (g/L: peptone, 10.0; yeast extract, 5.0, NaCl, 10.0, pH 7.2) containing increasing concentration of Co^2+^, Zn^2+^, or Cd^2+^ (0, 0.5, 1, 1.5, 2, 2.5, and 3 mM) and incubated for 48 h at 30°C (Wiegand et al., 2008). The lowest concentration of metals that inhibited growth (OD_540 *nm*_) completely was considered as MIC. The activity (at 10 h) of catechol 2,3-dioxygenase (C23DO, an enzyme catalyzing *meta*-ring cleavage of catechol in naphthalene metabolism) was monitored in the cell-free supernatant spectrophotometrically by measuring an increase in the absorbance at 375 nm due to the formation of 2-hydroxymuconic semialdehyde (_375_ = 33,000 M^–1^ cm^–1^) as described by Basu et al. (2006). Protein was estimated by the Bradford method using BSA as the standard (Bradford, 1976). All experiments were performed thrice in duplicates and the mean with the standard deviations are tabulated.

### Functional Analyses of Cargo Operons of ICE*nah*CSV86

In order to assess the functionality of the cargo operons of ICE*nah*CSV86, the promoter regions present upstream of the *nah* and *sal* operons were predicted using BPROM and SAPPHIRE, whereas the NahR binding site was predicted using multiple sequence alignment (for details, refer to “Supplementary Material”). The polycistronic nature of the *nah* and *sal* operons was validated using co-transcription analysis (refer to “Supplementary Material” and Supplementary Figure 2). In order to validate the functionality and strength of the predicted promoters and the NahR regulator; P*nah*, P*sal*, and P*sal*/*nah*R promoters were fused to gene (mcbC) encoding 1-naphthol 2-hydroxylase (1NH) as a reporter gene in pSEVA and the expression of 1NH was tested in *E. coli* BL21 (DE3) (Supplementary Figure 3). The details of the construct strategy and methodology are presented in the “Supplementary Material.”

## Results and Discussion

### *Pseudomonas bharatica* CSV86^T^ Genome Harbors Three Genomic Islands

**FIGURE 1:**
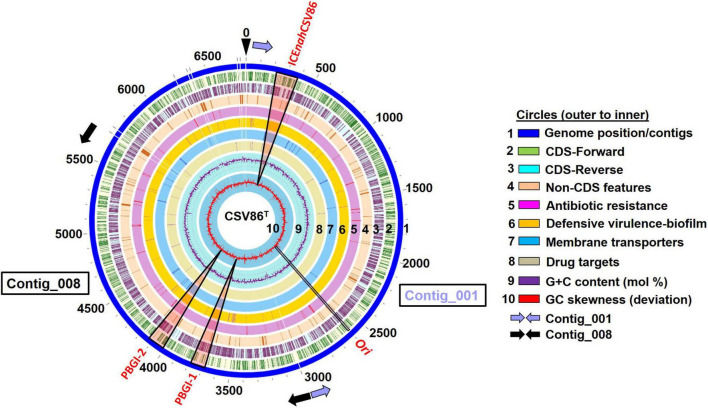
Circular genomic map (0–6,975 Kb) depicting the location of three genomic islands (GIs), predicted to be integrative conjugative elements (ICEs: ICE*nah*CSV86, PBGI-1, and PBGI-2 in the genome of *Pseudomonas bharatica* CSV86^T^ along with various genomic attributes (GC mol%, skewness, CDS features). The genome map was reconstructed using PATRIC 3.7.2.2. Elongated triangular boxes (light red color) indicate the positions of three GIs on the genome. *Ori*, the origin of replication and replicative gene catamers are depicted by black rectangle. The start and the end positions of Contig_001 and Contig_008 are depicted by light blue and black filled arrows, respectively.

**FIGURE 2:**
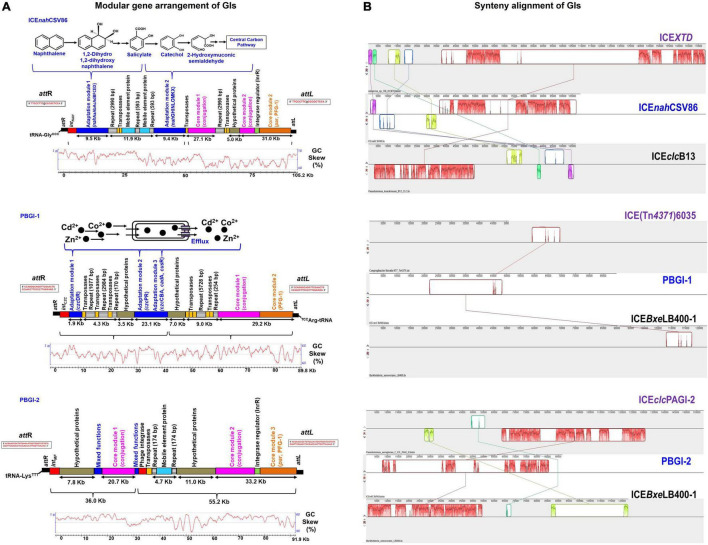
Comparative analyses of the three full-length GIs: ICE*nah*CSV86, PBGI-1 and PBGI-2 from *Pseudomonas bharatica* CSV86^T^. **(A)** Structural/modular organization of the three GIs, suspected to be ICEs. The color codes and depiction are as follows: cargo gene modules (blue), integration/excision (red), integrase regulator (green), conjugation (purple), partition modules (orange), regions containing repeats (lined boxes), genes encoding transposases (yellow), hypothetical proteins (gray), and mobile element proteins (light blue). Flanking *att*L and *att*R sites are indicated at the extreme ends (black solid rectangles) of the GIs and its sequences are depicted within the rectangular boxes. The functions encoded by respective cargo modules are depicted above the gene arrangement. The GC disparity plots (mol% skewness/deviations) for respective GIs are indicated below the gene arrangement. **(B)** Synteny alignment plots of predicted ICEs with the nearest similar ICEs from other bacteria obtained using progressive MAUVE alignment method. The blocks in different colors indicate linear conserved blocks (LCBs) and criss-cross connecting lines indicate the homologous LCBs amongst ICEs. For gene annotation and ORF details, refer to Supplementary Tables 3–5.

An advanced version of the draft genome of *P. bharatica* CSV86^T^ (AMWJ02000000: 6.79 Mb, 99.9% completeness, 0.03% heterogeneity) revealed the presence of three GIs of greater than 50 kb size on two of the largest contigs (Contig_001 and Contig_008, Figure 1). BLAST-based bidirectional hits of GIs against experimentally validated and genomically predicted ICE families in the ICEBerg 2.0 database predicted these GIs to be discrete, complete, and intact ICEs (Figure 1). Presence of genes encoding: (i) tRNA at either of the extreme ends of ICEs, (ii) integrase, and (iii) VirB4 homologs (hallmark gene of T4SS) having the highest similarity (72–95%) to ICE*clc* (ICE*clc*B13, ICE*XTD*, ICE*Bxe*LB400-1) confirmed affiliation of these elements to mating-pair type T4SS-ICEs (Wozniak et al., 2009; Poulin-Laprade et al., 2015). Genome analyses indicated that all three predicted ICEs in strain CSV86^T^ were present as a single copy. These elements were predicted to have no origin of replication (*ori*C). A single *ori*C site (831 bp with eight 9-mer DnaA boxes, constituting 48.01% AT content = 6,355,999 AT disparity, on contig_001) (Supplementary Figure 4) was found elsewhere in the genome of the strain CSV86^T^. Phylogenetic analysis of *ori*C and its associated elements indicated its lineage to non-ICE bearing members (*Pseudomonas japonica* WL, *Pseudomonas parafulva* CRS01-1) (Supplementary Figure 4). Further, the absence of any plasmid in strain CSV86^T^ confirms the chromosomal nature of these GIs (Basu and Phale, 2008). Similar to T4SS-ICEs, these elements were found to encode the main core gene modules harboring integrases, T4SS-T4CP, and DNA partitioning cluster (*par*, PFG-1) (Figure 2A). These three elements: ICE*nah*CSV86 (∼105 Kb), *Pseudomonas bharatica* genomic island-1 (PBGI-1) (∼89.8 Kb), and PBGI-2 (∼91.9 Kb) were found to encode 127, 112, and 136 RAST subsystem features, respectively. Amongst these, ICE*nah*CSV86 has been reported to be successfully transferred through conjugation to a recipient, *S. maltophilia* CSV89 with a frequency of 7 × 10^–8^ (Basu and Phale, 2008). Thus, this element has been classified as a probable ICE (ICE*nah*CSV86), based on the nomenclature system proposed (Burrus et al., 2002; Liu et al., 2019). A minor average G + C skew was observed in ICE*nah*CSV86 (62.5 mol%), i.e., close to chromosomal G + C content (63.2 mol%) of strain CSV86^T^. While significant deviations (ΔG + C = 9.3 and 7.13 mol%) were observed for PBGI-1 (G + C content = 53.9 mol%) and PBGI-2 (G + C content = 56.07 mol%), respectively (Table 1 and Figure 2A). A low synteny at the core gene modules and no synteny for cargo modules were noted for ICE*nah*CSV86 vs. PBGI-1 and PBGI-1 vs. PBGI-2 (Supplementary Figure 5A). Whereas ICE*nah*CSV86 and PBGI-2 shared moderate synteny at the core gene level (Supplementary Figure 5B). It has been established that genes (encoded on any MGEs, like GIs, including ICEs) originate from a common ancestor during specification events and are usually syntenic between closely related species, hence orthologous in nature. Sequences that show greater divergence from other species are more likely to perform distinct functions (Fang et al., 2010; Xu et al., 2019). Therefore, genes encoded on ICEs were analyzed for orthologous clusters using the OrthoVenn2 platform and annotated through the COG database with the WebMGA server (data not shown). A total of 83 orthologous clusters for ICE*nah*CSV86 were observed, while ICE*clc*B13 formed the maximum, i.e., 126 clusters (Supplementary Figure 6). Both PBGI-1 and PBGI-2 showed the highest number of singletons/unique clusters (*n* = 94), which were categorized into COG classes of unknown function/hypothetical proteins (I), general function (S), transcription (T), and defense mechanisms (V). Overall, a higher number of integrases (*n* = 3), hypothetical proteins (77), and conjugative proteins (9) were observed in PBGI-2, while a higher number of repeats (5) and transposases (8) were observed in PBGI-1.

### Genomic Islands in Strain CSV86^T^ Encode Metabolic and Adaptive Traits for Its Survival

#### ICE*nah*CSV86

ICE*nah*CSV86, a probable ICE, is located on the contig_001 (length 105,221 bp; nucleotide positions: 274,076 to 379,297) and has tRNA-Gly*^CCC^* gene (74 bp) at 5′-end. The 18 bp sequence at the 3′-end of tRNA-Gly (TTCCCTTCGCCCGCTCCA) forms the *att*L site (direct repeat), while the *att*R site (18 bp long, TTCCCTTCGCCCACTCCA) is located at 3′-end of ICE*nah*CSV86 with a single base substitution (A with G), defining the structural borders (Figure 2). The gene annotation of ICE*nah*CSV86 is summarized in Supplementary Table 3 and Supplementary Figure 7A. This element harbors typical T4SS core modules and cargo genes responsible for naphthalene metabolism. Strain CSV86^T^ utilizes naphthalene as the sole source of carbon and energy (Mahajan et al., 1994). Genes encoding enzymes for naphthalene metabolism were found to be a part of this element only and not elsewhere on the genome, hence designated as ICE*nah*CSV86. It showed the highest synteny with ICE*clc* elements [ICE*clc*B13 (102.7 Kb) and ICE*XTD* (173.7 Kb)]. ICE*nah*CSV86 exhibits a dual structure, i.e., characteristic of both ICE*clc*B13 and ICE*XTD*. However, unlike ICE*clc*B13, which is present in dual copies (Ravatn et al., 1998b), ICE*nah*CSV86 is present as a single copy, based on the genome analyses. ICE*nah*CSV86 displayed 76–83% nucleotide identity with core modules of various ICE*clc* members and displayed nearly similar gene synteny as observed in ICE*XTD* (Figure 2B). ICE*nah*CSV86 possesses a single *ori*T at position 41,464 to 41,838 Kb (375 bp), adjacent to an open reading frame (ORF) encoding SNF2 superfamily II DNA/RNA helicase (Supplementary Table 3). The *ori*T showed the highest homology with *ori*T of ICE*XTD* (92.8% identity) and ICE*clc*B13 (67.5%). Signature repeat motifs of 6 bp long (CCCTTT or GCCTTT, each 2 motifs) in *ori*T of ICE*nah*CSV86 were observed and found to be identical to ICE*clc*B13 (Supplementary Figure 8). The frequencies of repeats at nucleotide positions from 41,464 to 41,838 bp were 20.8- and 45.4-fold higher, respectively, than their random distribution on whole ICE*nah*CSV86, thus implying their recognition by helicase and *ori*T-processing proteins for DNA unwinding and conjugative transfer (Lawley et al., 2004). The presence of *ori*T and associated elements as a part of ICE*nah*CSV86 supports the successful conjugative transfer of naphthalene degradation property to *S. maltophilia* CSV89 (Basu and Phale, 2008).

Synteny alignment showed the occurrence of substantial rearrangement events (deletions and insertions) in ICE*nah*CSV86 as compared to ICE*clc* relatives (Figure 2B). Adjacent to tRNA-Gly*^CCC^*, gene encoding integrase (*int*NAH) responsible for catalyzing the site-specific integration and excision of ICE*nah*CSV86 was present (Supplementary Table 3). Similar to ICE*clc* (ICE*clc*B13), where integrase and its regulatory module are located at the two extreme ends of the ICE (Sentchilo et al., 2009), in ICE*nah*CSV86, InrR, an integrase regulator along with a DNA binding protein was found to be present ∼100.8 Kb downstream of *int*NAH and is likely to be involved in the regulation of *int*NAH expression (Figure 2A, Supplementary Table 3, and Supplementary Figure 7A). In strain CSV86^T^, naphthalene is metabolized to salicylate (upper pathway; *nah* operon: *nah*ABFCED), which is further channeled to TCA cycle intermediates *via* catechol (lower pathway; *sal* cluster: *nah*GHINLOMKX; Figure 2A). These cargo genes [*nah* (9.5 Kb) and *sal* (10.7 Kb) clusters] were found to be located on the coding strand of ICE*nah*CSV86, while *nah*R (encoding LysR type transcription regulator, which regulates both clusters) is located on the complementary strand. Most of the core genes (∼68 Kb) of ICE*nah*CSV86 are present on the complementary strand, except for some hypothetical proteins (Figure 2A and Supplementary Figure 7A). ICE*nah*CSV86 harbors core genes: VirB4, ∼19 Kb downstream to *nah*-cluster, murein hydrolase, and VirD4 at ∼42 Kb downstream of *nah* cluster (Supplementary Table 3). These three key proteins: murein hydrolase (degradation of the peptidoglycan for forming mating channel), VirB4-ATPase (DNA transfer by T4SS), and VirD4 coupling protein (DNA transfer by mating channel) are reported to be the essential components for ICE transfer (Christie et al., 2014). The ParAB locus in the PFG-1 core gene cluster was found to be present ∼66 Kb downstream of the *att*L site in ICE*nah*CSV86. This locus is reported to be involved in stabilization and the maintenance of ICEs (Bignell and Thomas, 2001). The entire core module (T4SS, T4CP, and PFG-1) of ICE*nah*CSV86 formed a large synteny with ICE*XTD*, while no synteny was observed for cargo modules. In ICE*nah*CSV86, insertion of the repeat (2,990 bp), transposases (1.12 Kb), and the stretch of hypothetical proteins (5 Kb) segregated the core module (67.2 Kb) into two sub-modules: Core-I (27.1 Kb) and Core-II (31.0 Kb). Similar insertion (of 2,990 bp repeat and 1.12 Kb transposase) along with an additional region comprising mobile element proteins and 593 bp repeat (in alternate) segregated cargo modules into “*nah*” and “*sal*” clusters (Figure 2A). BLASTn search of 2,990 bp repeats (of both cargo and core module) showed 99–100% identity with repeats of *Cycloclasticus zancles* 78-ME (obligate marine PAH degrading isolate), *Acinetobacter baumannii* (antibiotic resistant strains 7835 and 9201), and *Pseudomonas aeruginosa* strain AK6U. Interestingly, repeats found in both ICE*nah*CSV86 and *C. zancles* 78-ME genome encodes an ISL3-TnpA family transposase, guarded by Type-4 pilus assembly proteins. The 593 bp repeat harboring IS5 family transposase showed the highest similarity (99–100%) to probable ICE-bearing naphthalene degrading *Pseudomonas citronellolis* strain SJTE-3, isolated from waste sludge. Other transposases (IS66 and IS5) and mobile element proteins located on ICE*nah*CSV86 are affiliated to *Pseudomonas* spp. (95–99% similarity). An abundance of repeats and transposases within core and cargo modules serves as hotspots for possible recombination events and implied their acquisition from other organisms (Burrus, 2017). A significant deviation in GC skewness (Figure 2A, 12–13 GC mol%) of this stretch further supported the observation of the possibility of horizontal gene transfer events.

#### *Pseudomonas bharatica* Genomic Island-1

*Pseudomonas bharatica* genomic island-1 is located on the contig_008 (length 89,869 bp; nucleotide positions: 6,34,598 to 7,25,367), and has tRNA-Arg*^CCT^* gene (75 bp) at 3′-end followed by a repeat of 38 bp sequence that forms the *att*L site. An identical repeat is located at 5′-end (*att*R), thus defining the borders of this suspected ICE (Figure 2A). PBGI-1 is present as a single copy in the genome and its annotation is depicted in Supplementary Table 4 and Supplementary Figure 7B. Strain CSV86^T^ tolerates 2 mM concentration of various heavy metals (Co^2+^, Zn^2+^, Cd^2+^: CZC) supplemented during the growth of aromatics (Paliwal et al., 2014). Genes encoding efflux pump proteins/transporters for heavy metal cations (CZC) are found to be part of this GI as intact clusters, hence designated as PBGI-1. Compared to ICE*nah*CSV86, PBGI-1 exhibited a mixed structure, i.e., characteristic of more than one ICEs [ICE*clc*LB400 (122.2 Kb responsible for polychlorinated biphenyl metabolism), ICE*clc*CH34 (109.5 Kb for mercuric resistance), and ICE(Tn*4371*)6035 (50 Kb for acriflavine resistance)]. Synteny analysis of PBGI-1 showed linear conserved block with respect to a short stretch of core module (∼10 Kb) against ICE*clc*LB400 and ICE(Tn*4371*)6035 (Figure 2B). This element showed significantly poor similarity (<30%) with previously reported ICEs [ICE*Pae*LESB58-1 (*P. aeruginosa*), ICE*Cme*CH34-1 (*Cupriavidus metallidurans*), ICE*Hae*ULPAs1-1 (*Herminiimonas arsenicoxydans*), ICE*Axy*A8-1 (*Achromobacter arsenitoxydans*)] encoding heavy metal resistance (Cd, Zn, Co, Hg, As, and Cu). Overall, a different integration site (proximity to tRNA-Arg), the presence of hypothetical proteins within core modules, and Co-Zn-Cd resistance (*czc* operon) as a cargo function demarcated the uniqueness of this suspected ICE amongst reported ICEs for the heavy metal tolerance (Figure 2).

Downstream of tRNA-Arg, two core modules (conjugation and partition: T4SS, T4CP: a total of 29.2 Kb) were distributed conjointly on both the strands. Insertion of alternating repeats (sizes: 254 and 5,728 bp) and transposases (two Il-IS-2, size: 1,163 bp) separated the core modules from *czc* cargo modules (Figure 2A). Two membrane proteins (YraQ family) and ArsR transcription regulator were present upstream of these repeats and transposases (Supplementary Figure 7B). BLASTn searches (against RefSeq) of these observed repeats indicated 99–100% identity with repeats of *Cycloclasticus zancles* 78-ME (obligate marine PAH degrading isolate) and *A. baumannii* 7835 (antibiotic resistant), similar to ICE*nah*CSV86. These repeats were found to encode transposases (ISL*3*, TnpA family). Genes encoding Tn*7* transposition proteins (TsnBC) are located next to the repeats. These proteins catalyze transposition by promoting DNA bending to form a highly organized protein-DNA complex (Arciszewska and Craig, 1991). In the vicinity of this region, a stretch of hypothetical proteins (homologous to conserved hypothetical proteins of *Pseudomonas* spp.) was observed (Figure 2A). Like ICE*nah*CSV86, the cargo module (total ∼25 Kb) harboring genes for the tolerance of Co-Zn-Cd is located at the 5′-end (Figure 2A). Strikingly, PBGI-1 harbors all the reported systems (and the associated mechanisms) for Co^2+^, Zn^2+^, and Cd^2+^ resistance-efflux, which are localized into three sub-modules: (I) cation diffusion facilitator (CDF) CzcD based efflux cluster, (II) PIB4-type ATPase CzcP, and (III) HME-RND (Heavy MEtal-Resistance-Nodulation-Division)-driven CzcCBA cluster. Three alternating Il-IS-2 transposases (size: 560, 716, and 1,163 bp) followed by repeats (sizes: 1.077, 2.984, 170 bp), and stretch of hypothetical proteins segregated *czc* cargo clusters into two sub-modules (I and II). Module I comprising of *czc* regulator (*czc*R) and efflux resistance (*czc*D) showed the maximum homology (99%) with marine bacterial taxa, Module II comprising a single component transporting ATPase (*czc*P), and multi-component RND-resistance efflux pumps (*czc*ABC: transmembrane, membrane fusion, and outer membrane) showed affiliation with *Pseudomonas* spp. (Supplementary Table 4). This contrasting observation indicated probable differential gene acquisition events mediated through different sets of mobile elements. It is interesting to note that all of the mobile genes on PBGI-1, including repeats and transposases showed lineage to both aromatic degrading and metal-transforming halophilic marine bacteria (*Cycloclasticus zancles* 78-ME, *Halomonas axialensis, Salinicola peritrichatus*, and *Marinomonas* spp.) and *Pseudomonas* spp. (Supplementary Table 4). This signifies possible horizontal acquisition of mobile genes from these taxa. Codon-based *Z*-test for the selection also displayed diversifying selection (dN-dS vs. dS = 1.397, at 0.05% level) for *czc*DR. This observation indicates that in the presence of heavy metals (Co, Zn, and Cd), the tolerance pathway is likely to get stabilized with more non-synonymous substitutions than synonymous substitutions and tends to be retained in the population (Verma et al., 2014). A sigma factor RpoS and an integrase (*int*CZC) were found to be present upstream (5′-end) of the *czc* cluster (Supplementary Figure 7B). The RpoS is reported to be involved in making bistability decisions by the stationary phase cells to proceed for ICE transfer in ICE*clc*B13 (Miyazaki et al., 2012). It is also involved in activating promoters for regulated expression of integrase (*int*) and its regulator (InrR), thus mediating the high rate of ICE transfer to the recipient (Miyazaki et al., 2012). The presence of RpoS as a part of PBGI-1 could be attributed to regulating the expression of integrase in the absence of an integrase regulator (InrR).

#### *Pseudomonas bharatica* Genomic Island-2

The GI, *P. bharatica* genomic island-2 (PBGI-2) is also located on the contig_008 (length 91,907 bp; nucleotide positions: 947,073 to 1,038979) with tRNA-Lys*^TTT^* gene (75 bp) at 3′-end followed by a repeat of 61 bp sequence forming *att*L border and identical sequence repeat at 5′-end forms *att*R borders (Figure 2A). The PBGI-2 is present as a single copy in the genome and its gene annotation is summarized in Supplementary Table 5 and Supplementary Figure 7C. It showed structural similarity with ICE*clc* (ICE*clc*LB400), but maximum homology with GIs of *P. aeruginosa* (PAGI-5, PAGI-2 of *P. aeruginosa* C) encoding virulence, metal-resistance, and cytochrome biogenesis traits (Klockgether et al., 2007; Battle et al., 2009). Based on the maximum overall similarity observed with PAGI of *P. aeruginosa*, this GI is designated as PBGI-2 (Figure 2B). The core module (53.9 Kb) of PBGI-2 was found to be intact and segregated into three sub-modules (conjugation, *par*, and PFG-1) by two successive repeats of 174 bp flanked by mobile element proteins. Additionally, two long stretches of hypothetical proteins (7.8 and 11 Kb) are present upstream of the core modules (Figure 2A). Genes for DNA replication repair, membrane protein-TonB, short-chain dehydrogenase, methyl-accepting chemotaxis protein, disulfide isomerase, DNA-binding regulators, phage proteins, etc., were found to be present on PBGI-2, indicating mixed function nature of its cargo module. It was also found to harbor an additional P4 phage-type integrase and the associated regulator (InrR) near the 5′-end of the core module for regulating *int*PBGI-2 expression. BLASTN/P similarity searches showed a maximum homology of all cargo and core genes with *P. aeruginosa* strains (Supplementary Table 5).

The National Center for Biotechnology Information (NCBI) RefSeq-based homology search of PBGI-2 showed the presence of multiple copies of syntenic GIs in *Beta*- and *Gamma*-*proteobacteria* genomes. Whereas PAGI-2 type islands were reported in the genome of various (total of 31) *P. aeruginosa* and *P. putida* strains, of which 12 strains harbor one, 11 strains two, 7 strains three, and 1 strain with four PAGI elements. These are further grouped into 10 sub-types based on differential DNA-hybridization patterns (Klockgether et al., 2007). The presence of these ICE-like GIs in numerous taxa suggests that they probably form a family with a deep evolutionary origin. Based on the structural synteny with ICE*clc* members and higher homology with PAGI-2 of *P. aeruginosa* strain C, it can be hypothesized that PBGI-2 is an intermediate between an ICE and GI. Many PAGI elements are reported to excise spontaneously at a frequency of 10^–5^ to 10^–4^ (Rajanna et al., 2003; Lesic et al., 2004), although mutations, deletions, and genome rearrangements are likely to be responsible for the inability of such elements to achieve precise excision and mobilization. On the other hand, PAGI-2 does not spontaneously excise from its host chromosome during its growth *in vitro* and has been shown to transfer to other proteobacterial members at a low frequency (10^–5^ to 10^–4^) (Ravatn et al., 1998a; Klockgether et al., 2007). For other similar elements, like pKLC102 and PAPI-1, the frequencies of excision are of 1–3 orders of magnitude higher. Similarly, PBGI-2 harboring the phage-type integrase, plasmid-related genes, a type IV pilus biogenesis gene cassette, and a syntenic set of conserved ORFs similar to those detected in PAGI-2 and PAGI-3 indicate its ability to excise from the chromosome and form an extra-chromosomal circular intermediate during transfer.

### Selection Pressure Renders Integrative Conjugative Element Acquisition and Phylogenetic Divergence

**FIGURE 3:**
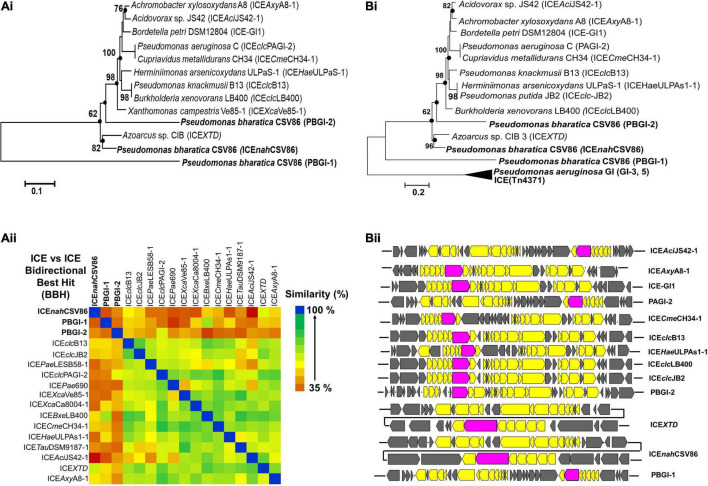
Evolutionary inferences and overall ICE related indices for three GIs, predicted to be ICEs from *Pseudomonas bharatica* CSV86^T^. **[A (i)]** Neighbor-joining (NJ) phylogenetic tree reconstructed using full-length nucleotide sequences of predicted ICEs and its nearest similar ICEs, and **[A (ii)]** Heat-map depicting bidirectional best hit-based average nucleotide identity (DNA–DNA homology level) of compared ICEs. **[B (i)]** Maximum-likelihood (ML)-based phylogeny of concatenated amino acid sequences of core gene modules (T4SS, T4CP), and **[B (ii)]** structural arrangement of core gene modules of various compared ICEs shown in **[B (i)]**. Trees were generated with 1,000 bootstrap iterations and the values at each node are represented as percentage replicates. The black solid circles represent generation of trees from all tree-making algorithms (NJ, ML, and ME).

Bidirectional alignment and phylogenetic analyses of ICE*nah*CSV86, PBGI-1, and PBGI-2 of strain CSV86^T^, as well as its modular components, were performed to delineate their ancestral root/origin and phylogenetic relatedness. ICE*nah*CSV86 formed a coherent cluster with ICE*XTD* as its nearest neighbor and corroborated the pairwise comparison data (Figure 3A). Interestingly, PBGI-2 was found to be a near-distant relative of ICE*clc* members, including ICE*nah*CSV86, whereas PBGI-1 deviated from all and formed a separate clade, as an out group [Figure 3A (i)]. Bidirectional hit-based DNA-homology (% similarity) data also demarcated ICE*XTD* to be the overall nearest relative of ICE*nah*CSV86, whereas, PBGI-1 and PBGI-2 were congruent with ICE*clc*LB400 (51% similarity) and PAGI-2 (48%), respectively [Figure 3A (ii)]. Phylogenetic analysis of integrases revealed their non-coherent nature, forming three distinct clusters (Supplementary Figure 9A). The *int*NAH formed a coherent association with integrases of *Pseudomonas, Betaproteobacteria*, and *int*bph-sal (cluster-I), whereas *int*PAGI-2 of *P. aeruginosa* C served to be a distant member. The *Int*PBGI-2 formed a monophyletic grouping with integrases of non-ICE Pseudomonads, whereas *intXTD* and *int*B13 of ICE*clc* were found to be the nearest ICE relatives. Unlike the other two, *int*CZC was a distant relative of all ICE-encoded integrases reported so far (Supplementary Figure 9A).

Phylogeny of concatenated core proteins (T4SS: VirB4, VirD4, TraG, TraI, and ParAB) showed two distinct branches. ICE*nah*CSV86 and PBGI-2 were very closely related and clustered with ICE*XTD*, while PBGI-1 was distantly related to ICE*clc* and others (Tn*4371*), thus forming a discrete branch [Figure 3B (i)]. The structural arrangement of T4SS core modules from various ICEs also corroborated the observation indicating a genetic coherence between ICE*nah*CSV86 and ICE*XTD*, while a mixed pattern was observed for PBGI-2 and PBGI-1 [Figure 3B (ii)]. Similar results were obtained for relaxases, where PBGI-1 remained out-cladded as a distant relative of all compared ICEs (data not shown). All three predicted ICEs from strain CSV86^T^ harbor a variable number of transposases, which are reported to be a key component for exchanging DNA under specific environmental conditions (Jurka et al., 2007). All these transposases were found to be distinct and showed a poor sequence homology (<30% similarity at 50% coverage) amongst each other. The transposases formed a mixed clustering (three distinct groups) and showed lineages with marine bacterial taxa (*Halomonas* spp., *Marinomonas*, and *Shewanella fodinae*) reported for encoding complex organic carbon metabolism and heavy metal resistance traits (Supplementary Figure 9B). Overall, phylogenomics and synteny mapping revealed that both ICE*nah*CSV86 and PBGI-2 might be speciated from a common ancestral lineage, i.e., ICE*XTD/LB4001-1* like ICE*clc* members. Codon-based *Z*-test and the rate of diversifying selection (dN-dS, dN/dS vs. dS) between syntenic core gene modules of ICE*nah*CSV86 and PBGI-2 showed a very high positive dN-dS values (4.07–8.85) (Supplementary Figure 5B), indicating a diversifying selection (non-synonymous substitution vs. synonymous substitution) of ICE*nah*CSV86 with respect to PBGI-2. With an intact and complete set of core modules and a conserved synteny of PBGI-2 with PAGI, we hypothesize that this element might be the precursor from which ICE*nah*CSV86 has been diverged after acquiring and stabilizing cargo genes (*nah-sal*) under selection pressure. The PBGI-1 might have undergone high degrees of recombination events, that have masked its evolution from a common ancestor. The presence of similar intermediary ICEs with higher recombination events has also been postulated (Wozniak et al., 2009; Baharoglu et al., 2013).

The presence of functionally active naphthalene degradation and Co^2+^, Zn^2+^, and Cd^2+^ transport-efflux genes on two different GIs in a single bacterium is a novel observation. Many naphthalene-degrading bacterial spp. are reported to harbor naphthalene metabolic clusters either on the plasmid (conjugative/non-conjugative) or transposon (Mohapatra and Phale, 2021). To date, no ICEs have been functionally characterized and reported for encoding naphthalene degradation traits. It is well established that aromatics including naphthalene and its derivatives have become ubiquitous in the environment (Duttagupta et al., 2020; Mohapatra and Phale, 2021). It has also been observed that many aromatic ring-hydroxylating dioxygenases like naphthalene dioxygenase (NDO, the first enzyme of the naphthalene degradation pathway) display “catalytic promiscuity” toward structurally related yet distinct compounds (Verma et al., 2019; Phale et al., 2020). Noticeably, such catalytic promiscuity of metabolic enzymes has been reported for a number of enzymes like biphenyl dioxygenase, benzoate dioxygenase, and toluene monooxygenase involved in the degradation of aromatics. Based on this information, it can be hypothesized that the ubiquitous presence of aromatics like naphthalene has imposed a substantial positive selection pressure on strain CSV86^T^ or similar spp. (from diverse biogeography) to acquire and evolve genes and enzymes for naphthalene metabolism through horizontal gene transfer events (by MGEs). Further, the genomic integration of these elements, re-organization, domain shuffling, and active-site optimization (for structurally related xenobiotic compounds) might have occurred to fine-tune the pathway, thus providing survival benefits to the recipient.

To validate this hypothesis, detailed analyses were performed for *nah* and *sal* clusters from genomes of naphthalene or aromatic degrading *Pseudomonas* spp. reported from diverse biogeographic origins (Table 2). Mega-BLAST analysis of *nah-sal* clusters of ICE*nah*CSV86 revealed similar and syntenic regions (of size 100–150 Kb, >70% coverage, 95% similar) in *P. citronellolis* SJTE-3, *Pseudomonas balearica* DSM 6083, *Pseudomonas benzenivorans* DSM 8628, *Pseudomonas bauzanensis* W13Z2, *Pseudomonas stutzeri* KOS6, etc. and some plasmids (pNAH7, pND6, pAK5, AN10, and pAS1) of *Pseudomonas* (Table 2).

**TABLE 2:** GC content and deviation (mol%, from genomic GC) of *nah* and *sal* cluster from different predicted ICEs and plasmids of various naphthalene degrading *Pseudomonas* spp. isolated from diverse biogeographic regions.

*Pseudomonas* spp.	Location of *nah-sal*	*nah* cluster GC mol% (Δ GC)*	*sal* cluster GC mol% (Δ GC)*	Isolation habitat	Country
*P. bharatica* CSV86^T^	ICE	52.06 (10.66)	62.03 (0.6)	Petroleum-spillage soil	India
*P*. *citronellolis* SJTE-3	ICE	52.05 (15.35)	62.30 (5.1)	Wastewater sludge	China
*P. stutzeri* ATCC 17588	ICE	52.7 (9.8)	62.03 (0.47)	Clinical	United States
*P*. *benzenivorans* DSM 8628	ICE	51.8 (13.4)	61.9 (3.3)	Oil-impacted groundwater	Golf Cost
*P. balearica* DSM 6083	ICE	52.18 (12.52)	62.11 (2.56)	Wastewater lagoon	Spain
*Pseudomonas* sp. MPDS	ICE	52.0 (8.7)	61.9 (−1.2)	PAH impacted soil	China
*P. stutzeri* 19SMN4	Plasmid	52.1 (10.9)	61.9 (1.7)	Wastewater lagoon	Spain
*P. veronii* Pvy	Plasmid	52.6 (7.4)	62 (−2.0)	Oil refinery sediment	Romania
*P. bauzanensis* W13Z2	ICE	53.85 (8.1)	61.94 (0.01)	Oil-impacted Bohai sea	China
*P. fluorescens* HK44	Plasmid	52.67 (5.18)	63.47 (5.12)	Gas manufacturing unit soil	United States
*P. stutzeri* AN10	Plasmid	52.09 (10.61)	60.83 (1.86)	Crude oil enrichment sediment	Mediterranean
*P. frederiksbergensis* AS1	Plasmid	53.02 (7.49)	62.91 (2.38)	As contaminated waste site	South Korea
*P. putida* AK5	Plasmid	53.6 (7.56)	–	Oil factory slime pit	Russia
*P. putida* NCIB 9816-4	Plasmid	53.11 (5.79)	63.59 (4.68)	Oil refinery soil	United States
*P. putida* ND6	Plasmid	53.11 (8.89)	63.41 (1.39)	Industrial wastewater	China
*P. putida* G7	Plasmid	53.7 (7.52)	63.30 (2.07)	PAH impacted soil	United States
*P. stutzeri* KOS6	ICE	53.88 (8.67)	62.82 (0.27)	Hydrocarbon sludge	Russia
*Pseudomonas* sp. K35	Plasmid	53.8 (8.45)	63.42 (1.15)	Coal-bed methane water	United States
*P. indoloxydans* JCM 14246	NIDN	–	64.81 (2.58)	Lindane impacted soil	India
*P. alcaliphila* JAB-1	ICE	–	65.4 (−2.9)	Impacted soil	Czechia
P. saponiphila DSM 9751	ICE	–	63.2 (1.0)	DSM collection	United States
*P. furukawaii* KF707	Plasmid	–	62.89 (2.51)	Impacted soil	Japan
*P. putida* B6-2	ICE	–	64.3 (−2.4)	Impacted soil	China

*ΔGC values in bracket with red font denote >5% G + C content deviation from respective genomic GC content indicating acquaintance of module from distant taxa, and those in blue denote G + C content deviation near-by/borderline of 5% deviation, and black denotes less than 5% G + C deviation indicating acquaintance of module from near-similar or similar species members. Positive values of ΔGC denote the net G + C mol% of the genome is higher than the cluster, while negative values indicate net G + C mol% of the genome is lower than the cluster. NIDN indicates Not Yet Identified.

**FIGURE 4:**
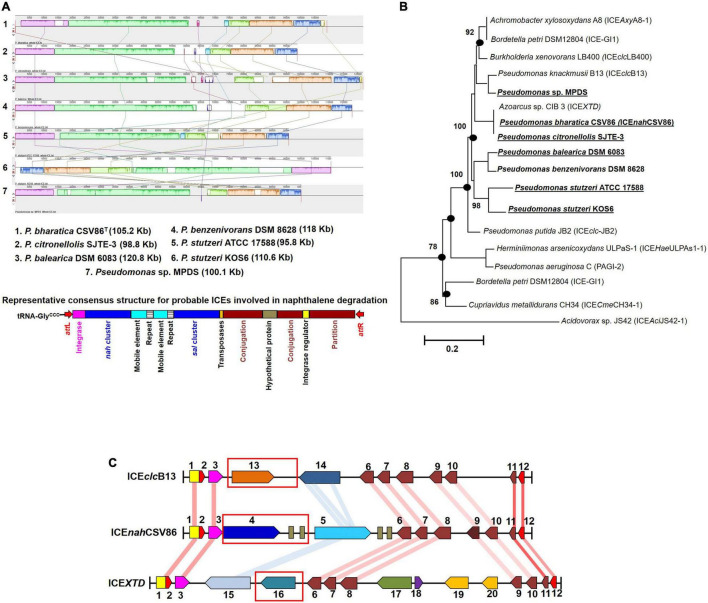
Comparative-functional genomic analyses of *nah*-*sal* clusters including adjacent up/down-stream sequences from various naphthalene/aromatic degrading *Pseudomonas* spp. **(A)** Synteny mapping of probable ICEs harboring *nah*-*sal* clusters and its comparison with ICE*nah*CSV86. The representative consensus structure for all naphthalene degradation encoding probable ICEs has been depicted at the bottom of the synteny plot. **(B)** ML phylogenetic analysis of ICE*nah*CSV86 with probable ICEs, belonging to the ICE*clc* family, from naphthalene and aromatic degrading bacteria. Taxa in bold indicate the probable naphthalene degrading bacteria while bold with underline indicate reported naphthalene utilization/degradation phenotype. Black dots at each node represent robustness of clade formed in all tree-making algorithms. Values near each branch of the phylogenetic tree indicate percent bootstrap re-iteration and bar below the trees indicate substitution (0.1–0.2%). **(C)** Artemis comparison-based synteny mapping of prototypical ICE*nah*CSV86 and its phylogenetically related neighbors (ICE*clc*B13 and ICE*XTD*). The connecting lines between LCBs indicate percent similarity between various modules: blue (<60%), light red (60–70%), and deep red (>70%). Rectangular red boxes represent the upper pathway cargo modules of the respective ICEs.

Interestingly, RAST annotation identified the presence of 5’ tRNA-Gly*^CCC^*, border repeats (*att*L/R), integrase as well as cargo genes (*nah*-*sal* clusters), and core modules, suggesting these genomic regions to be probable ICEs, similar to that observed in strain CSV86^T^ (Figure 4). The synteny alignment for all these hypothesized ICEs and ICE*nah*CSV86 indicated highly conserved cargo and core gene modules (as linearly conserved blocks, LCBs) (Figure 4A), thus denoting identical structural features, including *ori*T (homologous to ICE*nah*CSV86 and ICE*XTD*). Barring strain CSV86^T^ (Basu and Phale, 2008), none of them have been experimentally validated for conjugative transfer. Based on this analysis, we propose ICE*nah*CSV86 to be a prototypical element for naphthalene degradation with consensus features. A monophyletic and coherent clustering was observed for ICE*nah*CSV86 and all probable ICEs (Figure 4B). Further, a highly conserved consensus *att*L (except substitution of A with G in the last 13 bp in two members), coherent tRNA-Gly, and integrase (syntenic to ICE*nah*CSV86) supported ICE*nah*CSV86 to represent a prototypical element (Supplementary Figure 10). A modular synteny plot confirmed ICE*XTD* and ICE*clc*B13 as the nearest relatives, thus classifying these probable ICEs as members of ICE*clc* (Figure 4C). It is observed that the typical core and structural elements were highly syntenic (60–76% identical with LCBs) in all three ICEs. Out of two cargo modules (representing upper pathway and lower pathway), a moderate synteny (45–52% identical) was observed in the lower pathway of ICE*nah*CSV86, ICE*clc*B13, and ICE*XTD*. Interestingly, the upper pathway in all three ICEs: *nah* cluster in ICE*nah*CSV86, *clc* cluster in ICE*clc*B13, and *tod* cluster in ICE*XTD* were non-syntenic (<20% similar). Guarding these upper pathways, the presence of various mobile elements (multiple repeats and transposases) suggested a separate modular fusion of the upper cluster/pathway within the ICE frame.

**FIGURE 5:**
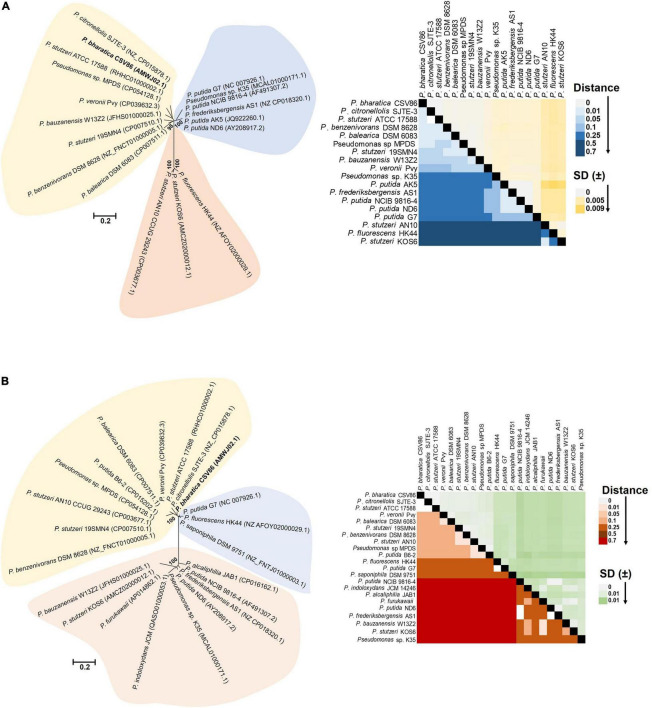
Phylogenomic analyses of cargo gene modules (*nah-sal* cluster) of ICE*nah*CSV86 and other mobile elements (probable ICEs, plasmids, transposons) from naphthalene degrading *Pseudomonas* spp. Phylogenetic trees of **(A)** *nah* cluster and **(B)** *sal* cluster are reconstructed using ML-based algorithm and validated using NJ, ME methods with 1,000 bootstrap iterations. The tree is optimized using interactive Tree of Life (iTOL), and the best optimum tree is depicted here. The color-shaded areas around the clades depict demarcation of differential cladding of clusters present on probable ICEs (light yellow), plasmids (light blue), and mixed elements (light pink, which include conjugative/non-conjugative transposons and intermediate forms). The heat-maps depict the *p*-distance based pairwise similarity of **(A)** *nah* clusters and **(B)** *sal* clusters located on various mobile genetic elements.

The *p*-distance based pairwise divergence estimate showed that *nah* cluster of ICE*nah*CSV86 has the highest homology with *P. citronellolis* SJTE-3, *P. stutzeri* ATCC 17588 (0.000 ± 0.001 distance), *P. benzenivorans* DSM 8628 (0.002 ± 0.001 distance), and *P. balearica* DSM 6083 (0.008 ± 0.002 distance) (Figure 5A). ML-based phylogenetic reconstruction corroborated this observation, where *nah* cluster of ICE*nah*CSV86 cladded with other probable ICE-bearing members and deviated from plasmid-encoded *nah* clusters as distant relatives (Figure 5A). Similar results were observed for *sal* cluster (Figure 5B). It is interesting to note that, a congruency/commonality was observed in terms of their habitat/ecotype, where all the probable naphthalene-ICE bearing members are isolated from oil, petroleum, or waste-impacted habitat, implying the role of the selection pressure; thus, helping strains to acquire and evolve these elements (Table 2). It has been reported that a broad GC content (>5%) change (distinct GC content of a cluster from the host genome) in stretches of prokaryotic genomes is indicative of the exchange of DNA/acquisition of gene(s) through HGT (Bohlin et al., 2010; Hildebrand et al., 2010). GC skewness analysis suggests that *nah* cluster displays higher order of mol% deviation as compared to *sal* cluster (Table 2). For *nah* cluster, the lowest deviation (5.18 mol%) was observed for *Pseudomonas fluorescens* HK44 and the highest (15.35 mol%) for *P. citronellolis* SJTE-3, whereas ICE*nah*CSV86 (10.66%) lied close to probable ICE of *P. stutzeri* AN10 (10.61%). On the contrary, the G + C mol% of *sal* cluster was significantly close to genomic G + C content, except for *P. fluorescens* HK44 (5.12% deviation). We have observed that the core genetic module of ICE*nah*CSV86 shows a GC content (62.95%) close to the genome (63.2% GC), whereas adaptation modules deviated sharply, i.e., module-1 (*nah*, 59.2% GC), module-2 (*sal*, 58.9% GC). Insertion of mobile elements (repeats and transposases) flanking *nah*-*sal* clusters further suggested the acquisition of cargo modules by different horizontal transfer events (Bosch et al., 1999; Hirose et al., 2021). To further validate these observations, dN-dS vs. dS (rate of non-synonymous over synonymous substitution) was performed. Positive dN-dS value (5.439–5.731) for *nah* cluster indicated higher non-synonymous substitutions than synonymous substitutions. This observation indicated its diversifying selection within the population and its still-evolving nature, which can be attributed to differential substrate selection pressure at impacted niches, i.e., naphthalene/structurally related compounds (oil-derived). Whereas lower dN-dS (2.139–2.247) of *sal* cluster indicated its intermediary/near-stable nature within the population. Additionally, the regulatory gene (*nah*R: LysR type regulators) of strain CSV86^T^ showed 100% similarity with *P. stutzeri* AN10 and phylogenetic closeness to other ICE-encoded NahR, out-cladding it from plasmid encoded members (Supplementary Figure 11). Interestingly, the presence of transposases upstream of *nah*R in both CSV86^T^ and AN10 and the absence of such gene in other plasmid-bearing members further confirms similar horizontal acquisition behavior.

It has been hypothesized that catabolic pathways for aromatic metabolism have evolved through the modular fusion of at least two to three components (upper, middle, and lower pathways routes). For example, catechol *meta*-cleavage route is predominant and homologous in many bacterial taxa and is predicted to fuse with lower operon gene module (generating TCA intermediates). Whereas upper (peripheral) pathway operon might have been acquired as a separate module at a later stage, thus increasing the catabolic versatility of the recipient strain. Further to fine-tune metabolism, regulatory elements were recruited from the evolved bacterial genome(s) and were modified subsequently to optimize the expression of the catabolic enzymes (Harayama, 1994; Gerischer, 2002). The G + C content and codon usage analyses of the *nah* operon of *P. stutzeri* AN10 support this modular fusion and recombination-based evolutionary theory. It has been shown that G + C content and the codon usage patterns of *P. stutzeri* AN10 are different for both the upper and lower naphthalene pathway genes. A similar pattern is also observed in strain CSV86^T^. The occurrence of non-coding (repeat-transposases) DNA region, flanking the conserved *nah* upper pathway has been suggested to be involved in the mobilization and further rearrangements of this entire catabolic module (Eaton, 1994). Overall, these observations strengthen our hypothesis of modular fusion of *nah* cluster in ICE*nah*CSV86 upstream to *sal* operon. The modular regulation of enzymes involved in aromatic degradation (benzyl alcohol, hydroxybenzyl alcohol, and other aromatics) has been demonstrated at the biochemical level in CSV86^T^ (Basu et al., 2003). Carbon source dependent metabolic studies also indicate that the degradation of aromatic alcohol and naphthalene involves two regulons (Basu et al., 2003; Mohan and Phale, 2017). Cellular respiration (nmol O_2_ consumed min^–1^ mg^–1^) studies of strain CSV86^T^ grown on naphthalene, salicylate, and glucose on substrate naphthalene, salicylate, and catechol showed differential induction of modules/metabolic regulons. Similarly, we observed the metabolite-dependent induction of three regulons for the metabolism of naphthalene-based pesticide Carbaryl in *Pseudomonas* spp. (Singh et al., 2013). These results indicate that the end metabolite generated from the upper operon induces the enzymes involved in the middle or lower operons in a cascading manner, achieving metabolic efficiency.

Integrative conjugative elements-encoded degradation or resistance traits in bacteria provide advantages over plasmid-encoded traits. ICEs are able to integrate into the host chromosome in a site-specific manner, resulting in stable phenotypic property. Further, this eliminates the need for constant replication and maintenance as well as inherent variability in the copy number, which is observed in most plasmids. In addition, ICEs are reported to excise and transfer to new host(s) in response to a variety of signals like RecA-dependent SOS response to DNA damage, secretion of signaling molecules from recipients, the growth phase of the host, and additional mechanisms related to the expression of cargo genes (Johnson and Grossman, 2015). Interestingly, ICEs are also reported to facilitate the transfer of other mobile elements, thus providing genetic bioaugmentation/plasticity in the community for better adaptability and evolution (Delavat et al., 2017; Liu et al., 2019).

### Functional Analyses of P*nah*/P*sal* Promoters From ICE*nah*CSV86

**FIGURE 6:**
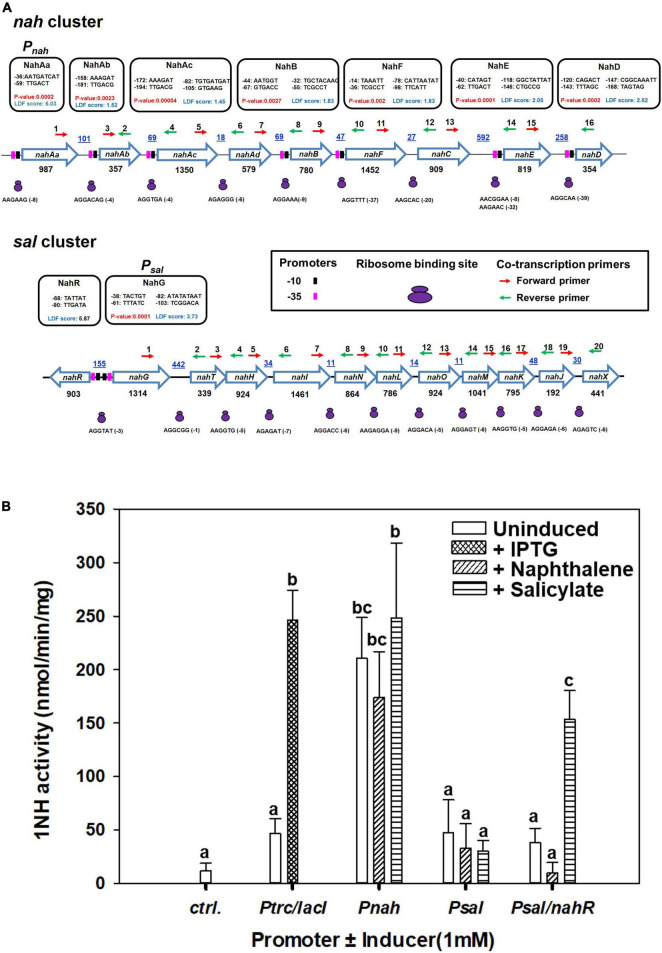
Analysis of the upstream regions of *nah* and *sal* operons present on ICE*nah*CSV86 of *Pseudomonas bharatica* CSV86^T^. **(A)** Operonic arrangement of upper (*nah*) and lower pathway *(sal)* genes and identification of putative promoter sequences [-10 (black filled rectangles) and -35 (pink filled rectangles) boxes], ribosome binding sites (RBS, as small and large purple ovals), and the location of primers [forward (red) and reverse (green) arrows] used for co-transcription analysis. The putative promoter sequences are depicted in the black box. The promoters with LDF scores have been predicted using BPROM and *P*-values were predicted using SAPPHIRE. The intergenic distances (bp) are indicated by blue color with underline, while gene lengths (in bp) are denoted below each gene. **(B)** Specific activity of 1-naphthol 2-hydroxylase (1NH) from various constructs expressed in *E. coli* BL21 (DE3) using different promoters and inducers. The bar represents the mean value with standard deviation (*n* = 3). Statistically significant differences (*P* = 0.05) among different promoter systems are denoted by a, b, c (Tukey’s *post hoc* test).

A strong promoter (designated P*nah*, LDF = 6.03, *P*-value = 0.0023) in the upstream and several weaker promoters throughout the *nah* cluster were predicted (Figure 6A). A single putative strong promoter (LDF = 3.73) could also be detected upstream of the *sal* cluster (Figure 6A). NahR, a LysR family transcription regulator (LTTR), is found to be located in between *nah* and *sal* cluster on ICE*nah*CSV86 with its own promoter (LDF = 5.87) and transcribed in the opposite direction of *nah* cluster. NahR binding site is found to be located 60 bp upstream of the transcriptional start site of *nah* and *sal* clusters (Supplementary Table 6). The promoters (-10 box and -35 box) and NahR binding site were found to be similar to other naphthalene degradation operons (Supplementary Figure 12). Co-transcription analysis confirmed the polycistronic nature of the *nah* and *sal* operons, thus validating the presence of strong promoters (P*nah* and P*sal*).

Amongst the promoters (P*nah*, P*sal*, and P*sal*/NahR) fused with 1NH, as a reporter gene, the activity from P*nah* was significantly higher than that of P*sal*, which showed expression only in the presence of NahR regulator and the inducer, salicylate (Figure 6B), while the expression from IPTG-induced P*trc* was similar to P*nah*. The *Psal/nahR* system has also been studied in both *Pseudomonas* and *E. coli* systems and has been found to be inducible (de Lorenzo et al., 1993; Calero et al., 2016),^11^ while the P*nah* has been reported to show leaky/constitutive expression (Burlage et al., 1990; Neilson et al., 1999). These observations provide an essential insight into the evolutionary logic driving the regulation of both operons. The presence of a strong but leaky promoter upstream to the upper *nah* operon is essential to generate the inducer (salicylate; the end product of the *nah* enzymes) at a sufficient concentration from the substrate (naphthalene). The lower operon harbors a relatively weak promoter which is tightly regulated and gets subsequently induced by a specific inducer metabolite generated by the action of upper pathway (*nah*) enzymes.

### Genomic Islands in *Pseudomonas bharatica* CSV86^T^ Confer Stable Phenotypic Traits

Strain CSV86^T^ grown in the absence of GI-encoded selection pressure for 60 generations did not depict a significant change in the naphthalene utilization property and heavy metal tolerance phenotype as compared to a culture grown under selection pressure. Specifically, the growth rate, cell biomass yield, or enzyme activity (catechol 2,3-dioxygenase) in naphthalene amended conditions were similar (Supplementary Table 7). The growth in the presence of heavy metals (Co^2+^, Zn^2+^, Cd^2+^) and MIC values also remained unaltered (Supplementary Table 7). Therefore, these GIs are stably integrated/maintained in the genome of strain CSV86^T^ and are not lost even in the absence of selection pressure. As a consequence, such elements have established themselves in diverse populations, playing an important role in bacterial evolution (Miyazaki et al., 2015; Delavat et al., 2017). Moreover, aromatic impacted habitats often contain a variety of toxic compounds like heavy metals, thus hindering the bioremediation process. Therefore, acquiring heavy metal tolerance trait is highly beneficial for a bacterium to survive in such contaminated niches (Jiménez et al., 2004; Phale et al., 2021). ICE*nah*CSV86 and PBGI-1 provide a survival advantage to strain CSV86^T^ in the presence of naphthalene and/or Co^2+^-Zn^2+^-Cd^2+^.

## Conclusion

*Pseudomonas bharatica* CSV86^T^ harbors three GIs (>50 Kb), suspected to be ICEs as single copies on the genome, belonging to the T4SS-ICE family: ICE*nah*CSV86 (105.2 Kb), PBGI-1 (89.8 Kb), and PBGI-2 (91.9 Kb), and are syntenic to ICE*clc* (ICE*XTD*), ICE*Tn4371*, and PAGI-2, respectively. Based on the cargo genes present on these GIs and phenotypic traits observed in strain CSV86^T^, ICE*nah*CSV86 was attributed for naphthalene degradation, PBGI-1 for heavy metal (Co^2+^, Zn^2+^, Cd^2+^) resistance-efflux, and PBGI-2 for mixed/unknown functions. ICE*nah*CSV86 is the only functionally and genomically characterized prototypical element (predicted to be an ICE) for naphthalene degradation with a low conjugative transfer frequency. PBGI-2 is hypothesized to be an intermediate element between ICE and GI and proposed to be an ancestor for ICE*nah*CSV86. The *nah* cluster is predicted to be integrated through modular fusion upstream to the *sal* cluster followed by rearrangement and domain reshuffling to achieve metabolic efficiency. These elements are stably integrated into the genome and are not lost even in the absence of selection pressure, rendering advantageous survival benefits and adaptive lifestyle to the strain CSV86^T^ under varied environmental conditions. The promoters of *nah*-*sal* on ICE*nah*CSV86 were transcriptionally active and found to be suitable for heterologous protein expression in *E. coli*. Functional genomic and genome-wide comparative analyses of multiple GIs in a single organism provide crucial insights into the evolution and distribution of metabolic traits, such as naphthalene degradation and metal tolerance through horizontal gene transfer, contributing to genomic plasticity, metabolic diversity, and niche colonization abilities to bacterial species. Further, the role of core modules in the acquisition of diverse cargo functions (as suited to the habitat) mediated through modular exchange and fusion has been hypothesized.

## Data Availability Statement

The datasets presented in this study can be found in online repositories. The names of the repository/repositories and accession number(s) can be found in the article/Supplementary Material.

## Author Contributions

PP, BM, and HM designed the study, performed data curation, validation, and interpretation, and agreed for the submission of the final version of the manuscript. BM performed the genome finishing, genomic data mining, comparative genomics, ICE-related experiments, and promoter prediction. HM conducted the promoter analyses, gene construct designing, co-transcription, and expression analyses. BM and HM wrote the manuscript drafts. PP managed the research funds and finalized the manuscript draft. All authors contributed to the article and approved the submitted version.

## Conflict of Interest

The authors declare that the research was conducted in the absence of any commercial or financial relationships that could be construed as a potential conflict of interest.

## Publisher’s Note

All claims expressed in this article are solely those of the authors and do not necessarily represent those of their affiliated organizations, or those of the publisher, the editors and the reviewers. Any product that may be evaluated in this article, or claim that may be made by its manufacturer, is not guaranteed or endorsed by the publisher.

## Footnotes

1
https://bioinfo-mml.sjtu.edu.cn/ICEberg2/index.php

2
https://mage.genoscope.cns.fr/microscope/home/index.php

3
https://www.pathogenomics.sfu.ca/islandviewer/

4
https://db-mml.sjtu.edu.cn/ICEberg/

5
https://phaster.ca/

6
https://orthovenn2.bioinfotoolkits.net/home

7
https://www.ezbiocloud.net/

8
http://tubic.org/doric/public/index.php/index/browse/bacteria

9
https://www.ezbiocloud.net/tools/orthoani

10
https://tygs.dsmz.de/

11
https://parts.igem.org/Part:BBa_J61051
