# Supplementary material for: Life Within a Contaminated Niche: Comparative Genomic Analyses of an Integrative Conjugative Element ICEnahCSV86 and Two Genomic Islands From Pseudomonas bharatica CSV86T Suggest Probable Role in Colonization and Adaptation
Source: Front Microbiol. 2022 Jul 6;13:928848. doi: 10.3389/fmicb.2022.928848 (PMC9298801; doi:10.3389/fmicb.2022.928848)
Supplement: Supplementary file 1 [file Data_Sheet_1.docx]

**Supplementary Material**

**(Revised Version, ID: 928848)**

**Life within a contaminated niche: Comparative genomic analyses of an integrative conjugative element ICE*nah*CSV86 and two genomic islands from *Pseudomonas bharatica* CSV86^T^ suggest probable role in colonization and adaptation**

Balaram Mohapatra, Harshit Malhotra, Prashant S. Phale^*^

Department of Biosciences and Bioengineering, Indian Institute of Technology-Bombay, Powai, Mumbai, 400076, India

***Address correspondence:** Prashant S. Phale; e-mail, pphale@iitb.ac.in

## A. Additional Materials and Methods

## PCR re-sequencing and validation of gaps, positions and integrity of suspected ICEs

To confirm the predictions and appropriate organization of three suspected ICEs in CSV86^T^, the first/ standard version of genomic draft (AMWJ01000000) was aligned with the advanced version (AMWJ02000000) using QUAST-Icarus [(http://cab.cc.spbu.ru/quast/)](http://cab.cc.spbu.ru/quast/). Set of primers (**Table S1**) were designed to amplify the gaps observed after aligning, as depicted in **Figure S1**. The gap regions were PCR amplified, products were gel purified using gel extraction SV kit (GeneAll) and sequenced. The amplicon sequences (both directions, forward and reverse) obtained were processed, true nucleotide positions were extracted and concatenated to obtain the desired contigs using BioEdit (version 7.0.5.3). The obtained amplicon sequences were identical to that of the advanced genomic draft.

**Functional analyses of the cargo operons of ICE*nah*CSV86**

**I. Promoter and NahR binding site prediction, and co-transcription studies**

Promoter analysis of *nah* and *sal* clusters located on ICE*nah*CSV86 was performed using the bacterial promoter prediction tools BPROM ([http://www.softberry.com/berry.phtml?topic=bprom)](http://www.softberry.com/berry.phtml?topic=bprom) and SAPPHIRE [(https://sapphire.biw.kuleuven.be/)](https://sapphire.biw.kuleuven.be/). Ribosome binding sites were predicted manually by considering AG-rich consensus sequences of proteobacterial class (Omotajo et al., 2015). Additionally, the NahR regulator-binding site was predicted in the upstream region of *nah* and *sal* operons based on the available literature and multiple sequence alignment.

To demonstrate the polycistronic nature of mRNA transcribed from *nah* and *sal* cluster, co-transcription analysis was performed. Total RNA was isolated from naphthalene grown cells (OD_540_=0.4-0.7) using RNeasy bacterial mini-kit (Qiagen, Germany) as per the manufacturer’s instructions. DNA contamination was eliminated by DNase I (2 U; Invitrogen, USA) treatment and RNA was quantified using Nano-Spec microplate reader (Multiskan Go; Thermo Fisher, USA). cDNA was synthesized using DNA-free total RNA (1 µg) as template and random hexamers as primers using Superscript c-DNA synthesis kit (Invitrogen, USA). The cDNA was used as the template for PCR reaction to detect the co-transcription of various genes from *nah*-*sal* operons using gene-specific primers, as shown in **Table S2, Figure S2**. A nested PCR reaction (3, 4, or 5 genes, **Table S2**) was set-up to confirm the transcript amplicons. Reaction with genomic DNA and without cDNA was performed as positive control and no template control, respectively.

**II. Cloning of *Pnah, Psal and Psal/nahR* into pSEVA234-1NH**

The expression vector pSEVA234, harboring the *trc/lacI^q^* promoter system was a kind gift from Dr. de Lorenzo’s lab and the SEVA collection team, CNB-CSIC, Spain. The gene *mcb*C encoding 1-naphthol 2-hydroxylase (1NH) was amplified using gene-specific primers (**Table S2**) from the genomic DNA of *Pseudomonas* sp. C5pp as template. PCR product of 1.8Kb was gel eluted, digested with *Xba*I/*Sal*I and cloned into pSEVA234 at *Xba*I/*Sal*I site to generate pSEVA234-1NH construct, and sequence confirmed using gene-specific primers. The 117 bp fragment (upstream of *nah* cluster) harbouring P*nah* was amplified using genomic DNA of strain CSV86^T^ as the template and the promoter specific primers (**Table S2)**. The amplified PCR product of 117bp was gel eluted, digested with *Pac*I/*Avr*II, and cloned in pSEVA234-1NH at *Pac*I/*Avr*II sites, thus replacing the original *trc/lacI^q^*promoter system. This construct, with insert size of 1.9Kb is referred to as pSEVAP*nah*-1NH (**Figure S3A**). Similarly, P*sal* (119 bp) and P*sal*/*nah*R (1058 bp) sequences (present upstream of *nah*G) were PCR amplified from the genomic DNA of strain CSV86^T^ using promoter specific primers (**Table S2**)**,** gel eluted, digested with *Pac*I/*Eco*RI, and cloned into pSEVA234 at *Pac*I/*Eco*RI sites, thus generating 1.9 kb and 2.8kb insert-based pSEVAP*sal*-1NH and pSEVAP*sal*/*nah*R-1NH constructs (**Figure S3A**)**.** These constructs were further sequence confirmed by 1^st^ BASE (Malaysia).

**III. Expression of 1NH in *E. coli* using P*nah* and P*sal* promoters**

Heterologous expression of 1NH was carried out in *E. coli* BL21(DE3) using constructs pSEVA234-1NH, pSEVAP*nah*-1NH, pSEVAP*sal*-1NH and pSEVAP*sal/nah*R-1NH expressing 1NH under *trc/lacI^q^*, P*nah*, P*sal* and P*sal*/*nah*R promoters, respectively (**Figure S3B**). *E. coli* BL21(DE3) was transformed with the respective constructs by CaCl_2_-based heat-shock treatment method (Sambrook and Russell, 2006). Transformants were selected onto Luria-Bertani (LB) agar supplemented with kanamycin (40 µg/mL). A single colony (for the respective construct having a promoter: *trc/lacI^q^*, P*nah*, P*sal* or P*sal*/*nah*R) was inoculated and grown overnight in 5 mL LB broth containing kanamycin. Actively-growing culture (200 µL) was transferred into 10 mL LB with kanamycin (40 µg/mL) and allowed to grow until culture OD_600_=0.5-0.6 at 37°C; followed by induction with either IPTG (1 mM for pSEVA234-1NH) or naphthalene or salicylate (1 mM for pSEVAP*nah*-1NH, pSEVAP*sal*-1NH and pSEVAP*sal/nah*R-1NH) for 8 h at 30°C. Uninduced *E. coli* BL21(DE3) cells were used as negative control. The cell-free extract (CFE) was prepared by sonicating cells in Tris-Cl buffer (50 mM, pH 7.5) followed by high-speed centrifugation as described in Swetha et al., (2007). The activity of 1NH was monitored from the CFE spectrophotometrically by measuring the decrease in the absorbance of NADH at 340nm (ε_340_=6220 M^-1^cm^-1^). Assay mixture (1 mL) contained appropriate amount of CFE, NADH (100 μM), FAD (6.25 μM), 1-naphthol (100 μM) and Tris-Cl buffer (50 mM, pH 7.5) (Swetha et al., 2007). Protein concentration was determined by the method of Bradford (1976) using BSA as the standard. Statistical analysis and graphical representation of 1NH specific activity (nmol/min/mg) in various samples was carried out using one way ANOVA, followed by Tukey’s *post hoc* analysis using SigmaPlot version 12.3, Systat Software, Inc., USA. Protein expression was analyzed by SDS-PAGE (Laemmli, 1970).

**References**

Bosch, R., Garcı́a-Valdés, E., and Moore, E. R. (1999). Genetic characterization and evolutionary implications of a chromosomally encoded naphthalene-degradation upper pathway from *Pseudomonas stutzeri* AN10. *Gene.* 236:149-157.

Bradford, M. M. (1976). A rapid and sensitive method for the quantitation of microgram quantities of protein utilizing the principle of protein-dye binding. *Analytic. Biochem.* 72:248-254.

Durante-Rodríguez, G., De Lorenzo, V., and Nikel, P. I. (2018). A post-translational metabolic switch enables complete decoupling of bacterial growth from biopolymer production in engineered *Escherichia coli*. *ACS Synth. Biol.* 7:2686-2697.

Huang, J. Z., and Schell, M. A. (1991). In vivo interactions of the NahR transcriptional activator with its target sequences. Inducer-mediated changes resulting in transcription activation. *J. Biol. Chem.* 266: 10830-10838.

Laemmli, U. K. (1970). Cleavage of structural proteins during the assembly of the head of bacteriophage T4. *Nature.* 227:680-685.

Omotajo, D., Tate, T., Cho, H., and Choudhary, M. (2015). Distribution and diversity of ribosome binding sites in prokaryotic genomes. *BMC Genomics.* 16:1-8.

Park, W., Jeon, C. O., and Madsen, E. L. (2002). Interaction of NahR, a LysR-type transcriptional regulator, with the α subunit of RNA polymerase in the naphthalene degrading bacterium, *Pseudomonas putida* NCIB 9816-4. *FEMS Microbiol. Lett*. 213:159-165.

Sambrook, J., and Russell, D. W. (2006). Preparation and transformation of competent *E. coli* using calcium chloride. Cold Spring Harbor Protocols, 2006(1), pdb-prot3932.

Schell, M. A., and Poser, E. F. (1989). Demonstration, characterization, and mutational analysis of NahR protein binding to *nah* and *sal* promoters. *J. Bacteriol.* 171:837-846.

Swetha, V. P., Basu, A., and Phale, P. S. (2007). Purification and characterization of 1-naphthol-2-hydroxylase from carbaryl-degrading *Pseudomonas* strain C4. *J. Bacteriol.* 189:2660-2666.

Tropel, D., and van der Meer, J. R. (2004). Bacterial transcriptional regulators for degradation pathways of aromatic compounds. *Microbiol. Mol. Biol. Rev.* 68:474-500.

**B. Supplementary Figures**

**Figure S1**


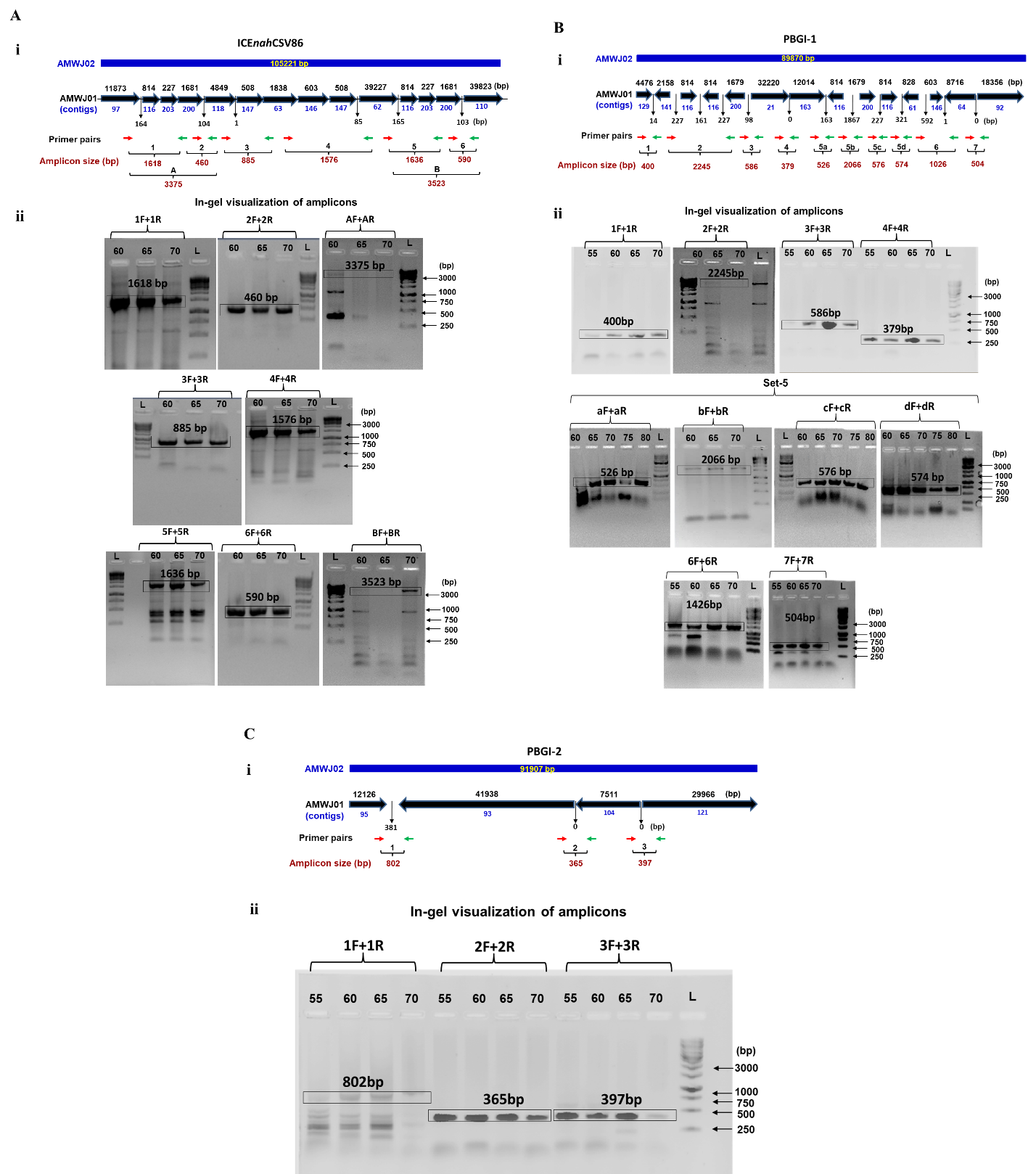


**Figure S1.** Alignment and mapping of advanced high-quality draft genome (version: AMWJ02000000) of *P. bharatica* CSV86^T^ harbouring three GIs, suspected to be ICEs (ICE*nah*CSV86, PBGI-1 and PBGI-2) with first/standard version of draft genome (AMWJ01000000) using BLAST aligner and Icarus contig viewer. The upper panel (i) in **A**, **B** and **C** denotes ordering and alignment of the contigs from standard draft (AMWJ01) with advanced draft genome (AMWJ02), as well as gaps (length in bp) along with primer pairs (red and green arrows) used for the amplification of contig positions spanning the gap regions. The lower panel (ii) in **A**, **B** and **C** depicts PCR amplified products with appropriate primers (see **Table S1**) by agarose gel electrophoresis. The desired amplicons (size in bp) are indicated within rectangular boxes along with the DNA ladder (as ‘L’).

**Figure S2**

**
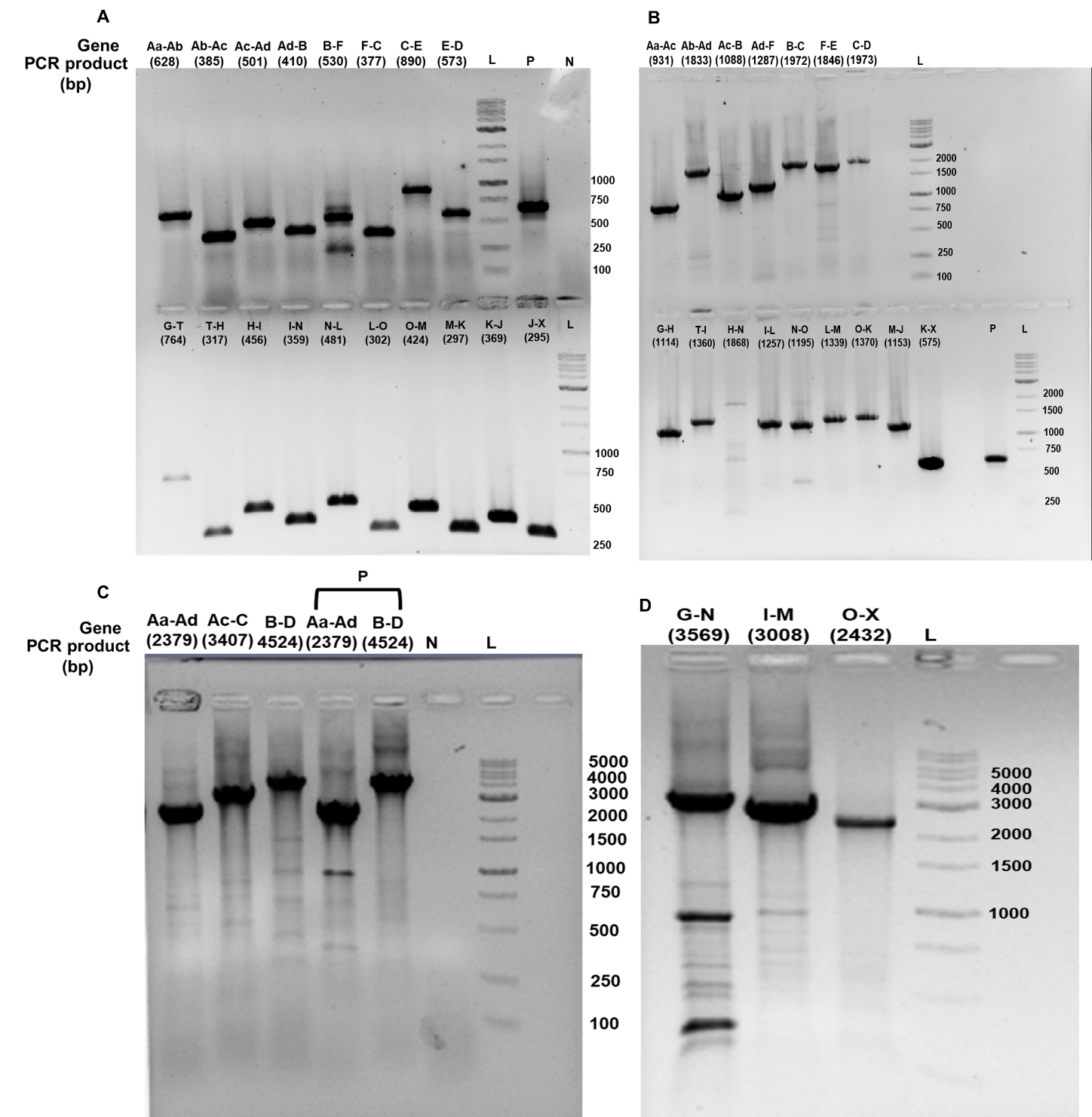
**

**Figure S2.** Co-transcription analysis of naphthalene metabolism clusters (*nah* and *sal* operons) in *P. bharatica* CSV86^T^. Agarose gel electrophoresis showing PCR amplification of overlapping regions of either (**A**) two, (**B**) three, (**C**) four, and (**D**) five neighboring genes using cDNA synthesized from the isolated RNA as template and appropriate primers (see **Table S2**). PCR with cDNA template and without reverse transcriptase was used as negative control (indicated as N) and PCR with genomic DNA of strain CSV86^T^ was used as positive control (indicated as P).

**Figure S3**

**A**

**
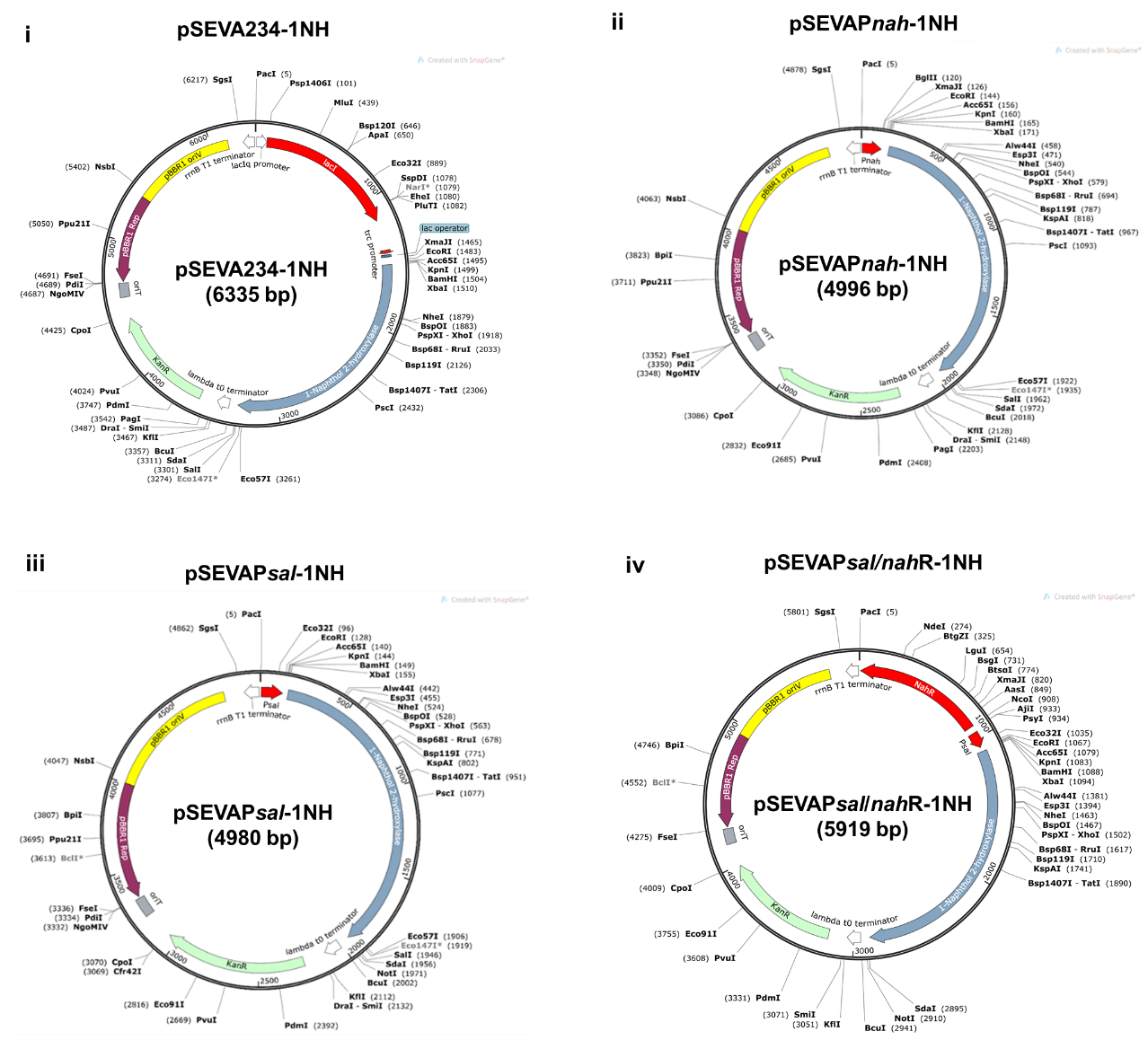
**

**
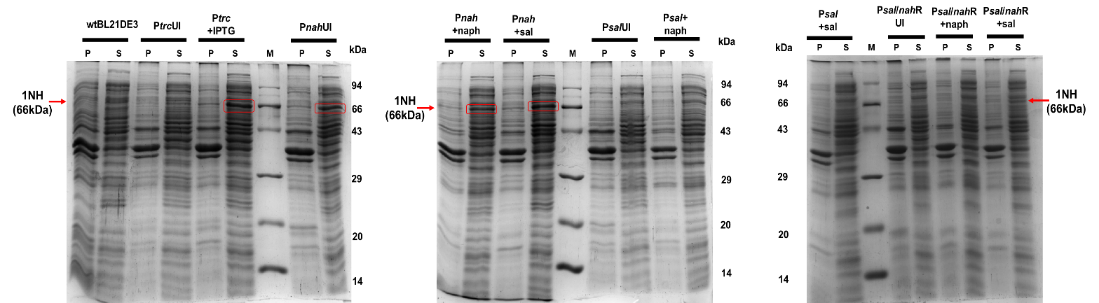
B**

**Figure S3.** Functional validation of the promoters of *nah*-*sal* operons present on ICE*nah*CSV86. **A)** Cloning of 1-NH, *Pnah, Psal* and *Psal/nahR* into pSEVA234 vector, yielding constructs: **i**) pSEVA234-1NH (with its original P*trc*/*lac*I^q^ promoter), **ii**) pSEVAP*nah*-1NH, **iii**) pSEVAP*sal*-1NH, and **iv**) pSEVAP*sal*/*nah*R-1NH. **B)** SDS-PAGE analysis of expression of 1-naphthol 2-hydroxylase (66kDa) from various promoter systems in *E. coli* BL21DE3 grown on LB in absence or presence of 1mM inducer (IPTG, naphthalene or salicylate). “P*trc*” denotes the *Ptrc/lacI^q^* expression system, “P*nah*” the *nah* promoter, “P*sal*” the *sal* promoter without regulator, “P*sal*/*nah*R” the *sal* promoter with NahR regulator, wtBL21DE3 was taken as a negative control. “P” represents the pellet fraction, “S” the soluble fraction, “M” the molecular weight marker, “UI” the uninduced samples while the inducers are mentioned as +IPTG, +naph (naphthalene) or +sal (salicylate). The band indicating 1NH (66kDa) is enclosed by a red box.

**Figure S4**

**
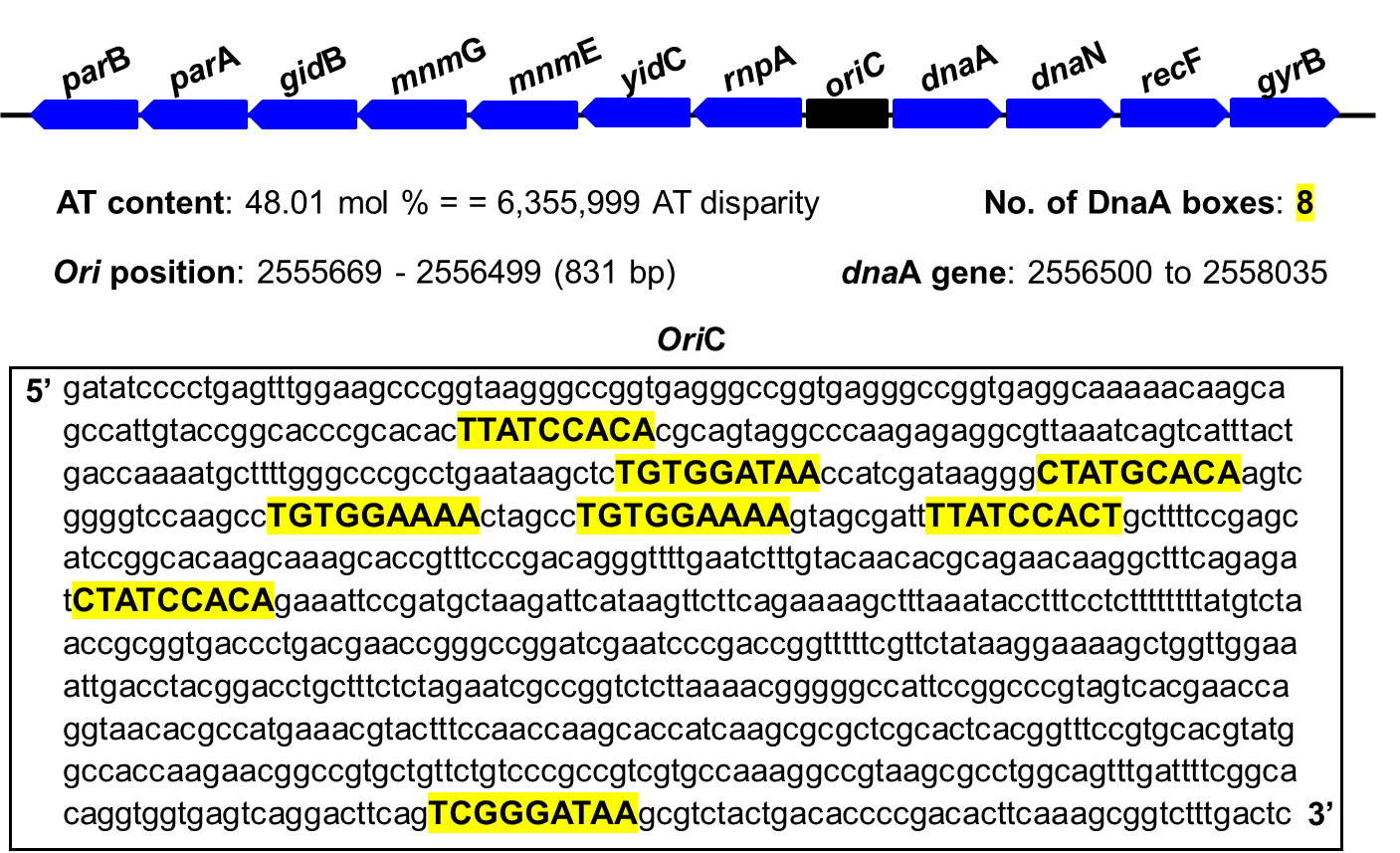
A**

**B**

**
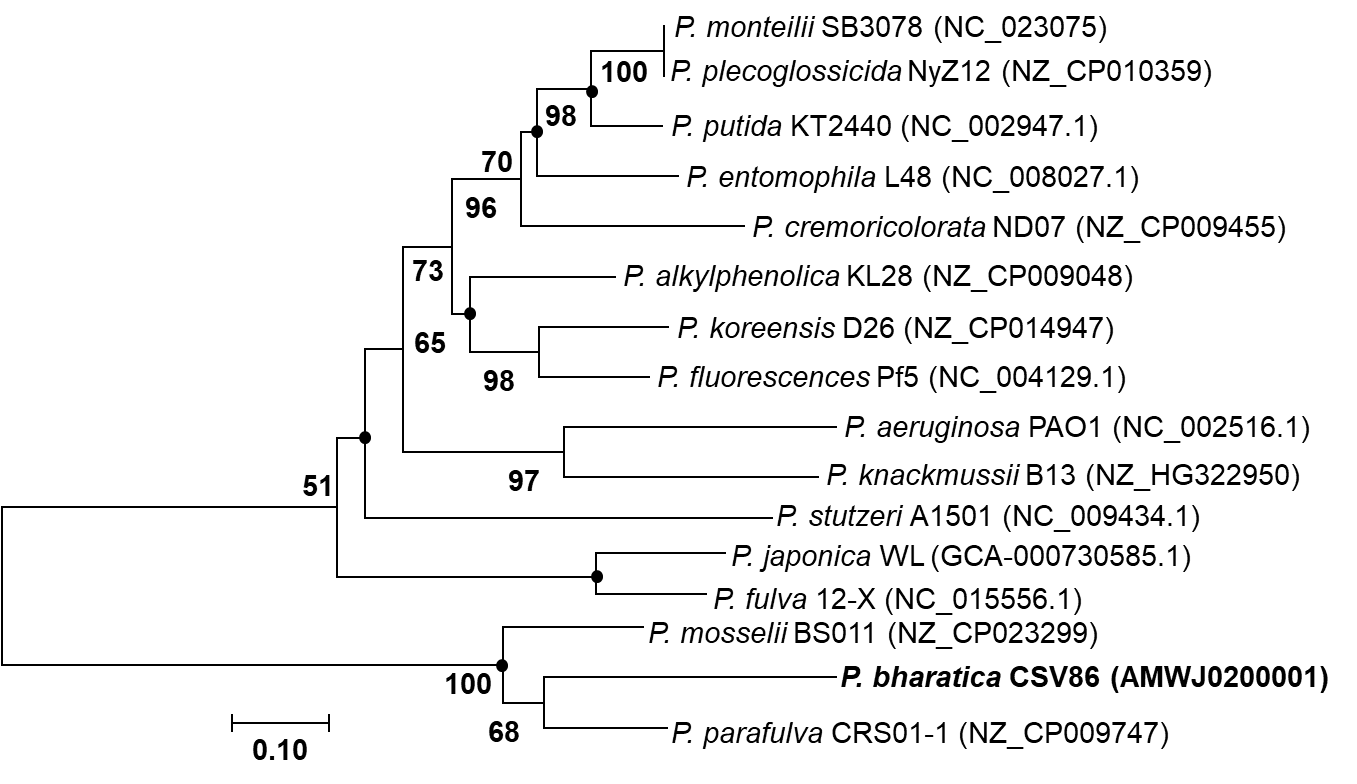
**

**Figure S4.** Genetic organization and phylogenetic analyses of genes involved in the replication of genome of *P. bharatica* CSV86^T^. **A)** Structural organization of *ori*C and its up- and down-stream elements, identification of *dna*A boxes and AT disparity at origin of replication in strain CSV86^T^. **B)** maximum-likelihood based phylogenetic reconstruction of concatenated replicative elements (*par*B-*gid*B-*yid*C-*rnp*A-*ori*C-*dna*A-*dna*N-*rec*F-*gyr*B). The tree was generated with 1000 bootstrap iterations using MEGA 7.0 and values at each node represent percentage replicates (%). The black solid circles represent generation of tree from all three tree making algorithms (NJ, ML, ME). The genomes of *P. knackmusii* B13 and *P. aeruginosa* PAO1 are taken as reference (RefSeq) genomes.

**Figure S5**

**A**

**
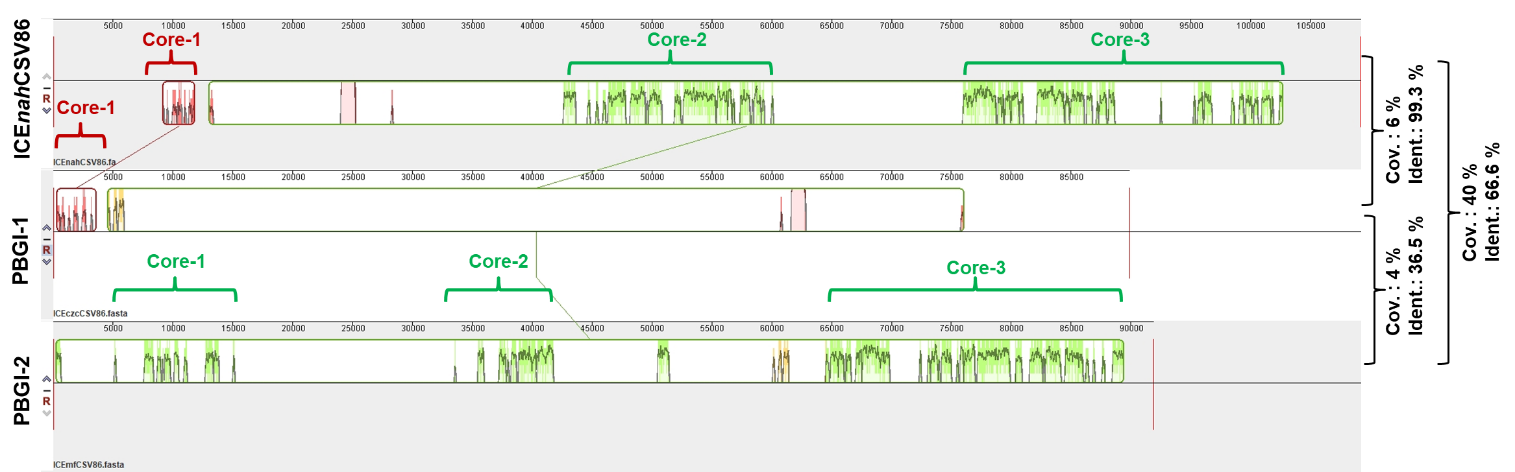
**

**B**

**
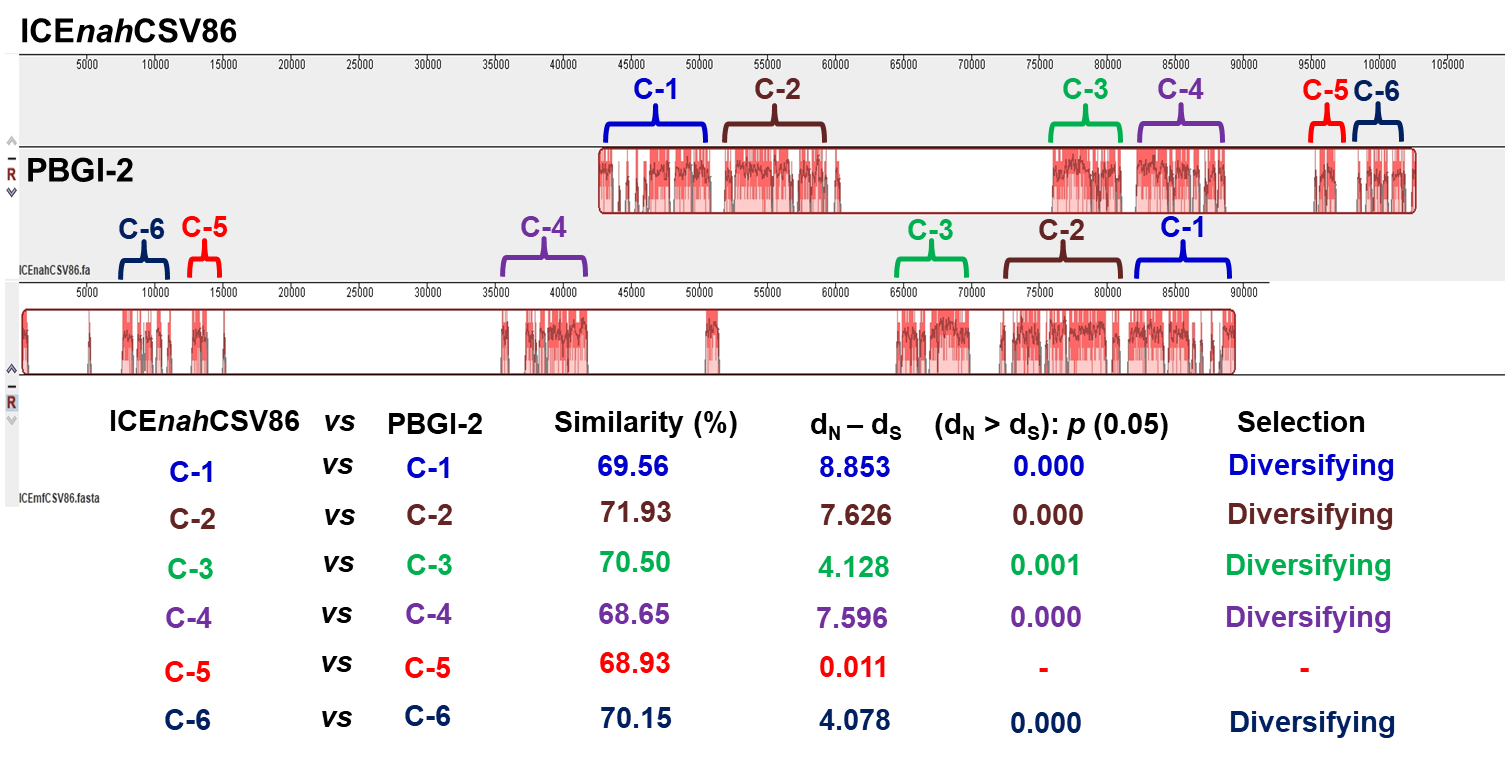
**

**Figure S5.** Synteny comparison of GIs from *P. bharatica* CSV86^T^ using MAUVE genome aligner with progressive alignment method. **A)** Synteny comparison amongst three suspected ICEs of *P. bharatica* CSV86^T^. **B)** Synteny plot along with homology (similarity %), and codon-based *Z*-test for selection (dN-dS) analysis between core gene modules of ICE*nah*CSV86 and PBGI-2. The blocks in different colours in (**A**) and red colour in (**B**) indicate linear conserved blocks (LCBs) and criss-cross connecting lines in (**A**) indicate the homologous LCBs. Similarity values (at nucleotide level) in (**A**) are presented at right side. The core regions in (**B**) are marked as C-1 to C-6 and statistically significant *p* values (at 0.05 % cut-off) are shown along with the dN-dS values.

**Figure S6**

**
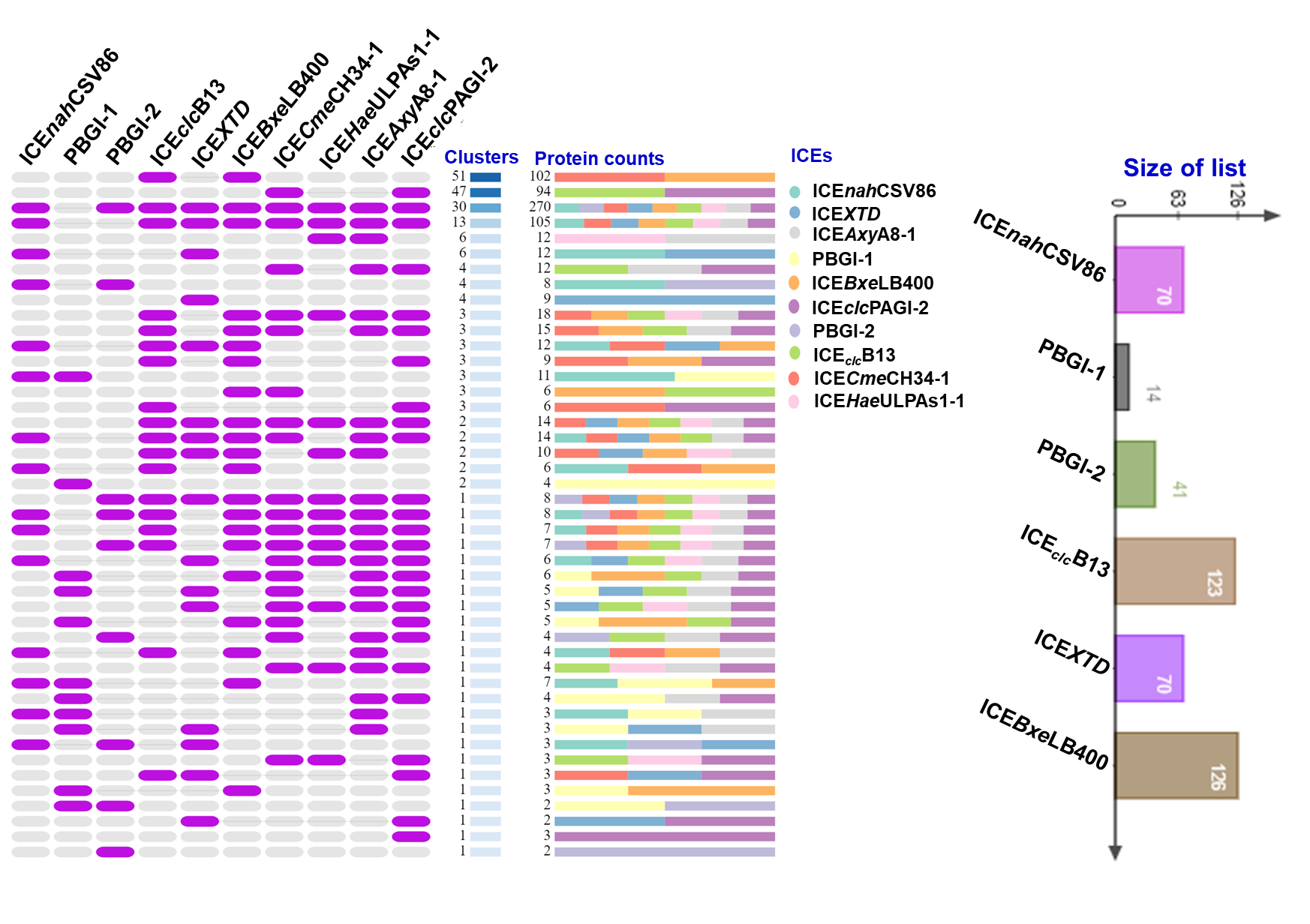
**

**Figure S6**. Ortholog clustering of genes encoded on three suspected ICEs from *P. bharatica* CSV86^T^ and comparison with other functionally and genomically characterized ICEs using OthoVenn2 platform. The coloured (purple) bars and abundance plot depict the clustering of different gene homologs into different orthologous groups. Bar graph indicates the size of each cluster amongst compared members.

**Figure S7**

**
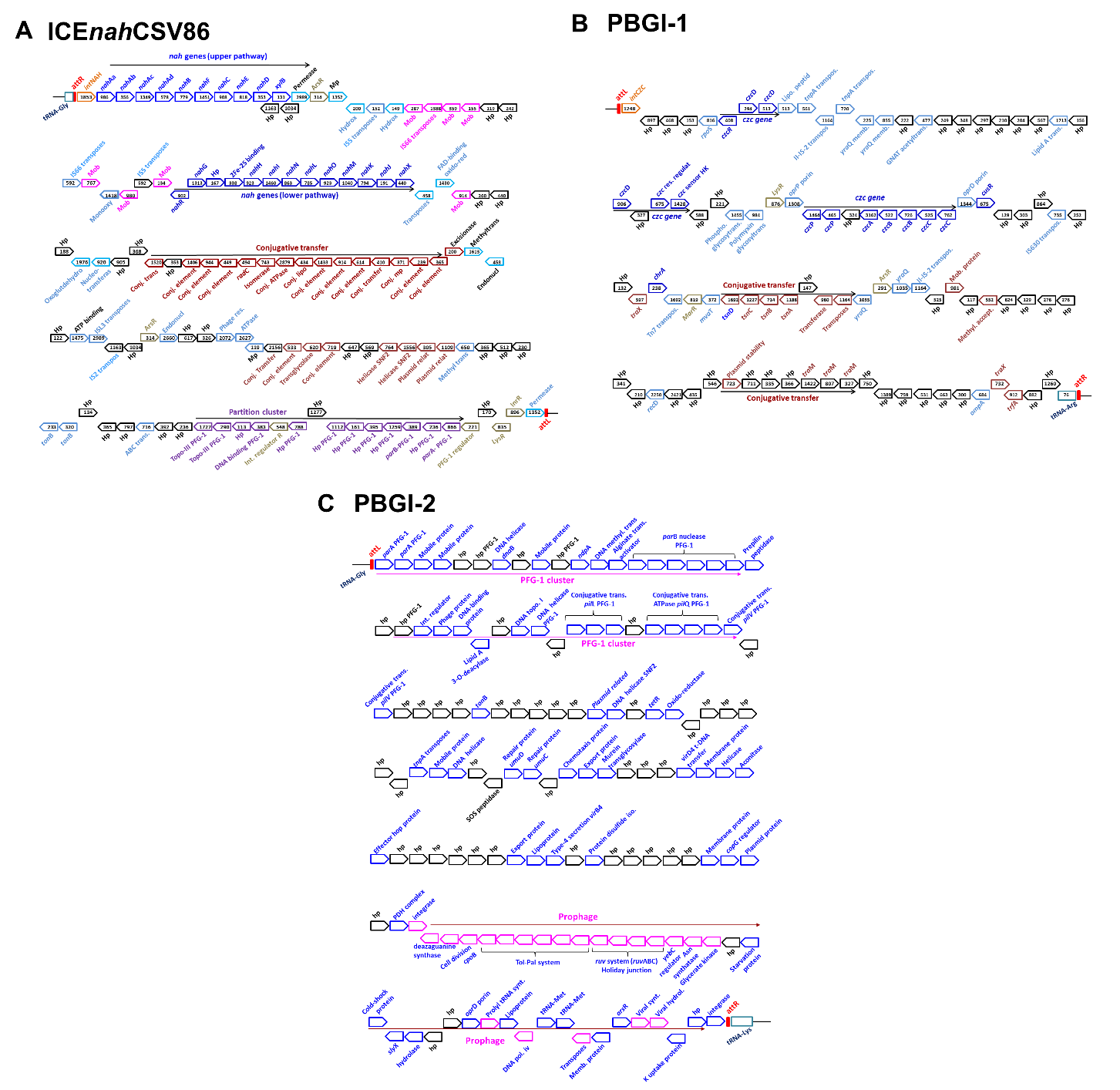
**

**Figure S7.** The linear genetic map showing gene arrangement of three suspected ICEs: **A)** ICE*nah*CSV86, **B)** PBGI-1, and **C)** PBGI-2 from *P. bharatica* CSV86^T^. Genes from coding and complementary strands are designated with right and left-oriented pentagons. Numbers within pentagon represent nucleotide length (in bp) of each gene (RAST-tk annotation). The core modules are coloured in brick red and purple pentagons, while cargo modules are coloured in blue. The hypothetical proteins (black), integrases (orange), regulators (olive tan), and other genes (cellular functions) (light blue) are denoted in respective coloured pentagons. The tRNAs and *att* sites are depicted in open-teal and filled-red rectangles, respectively.

**Figure S8**

**
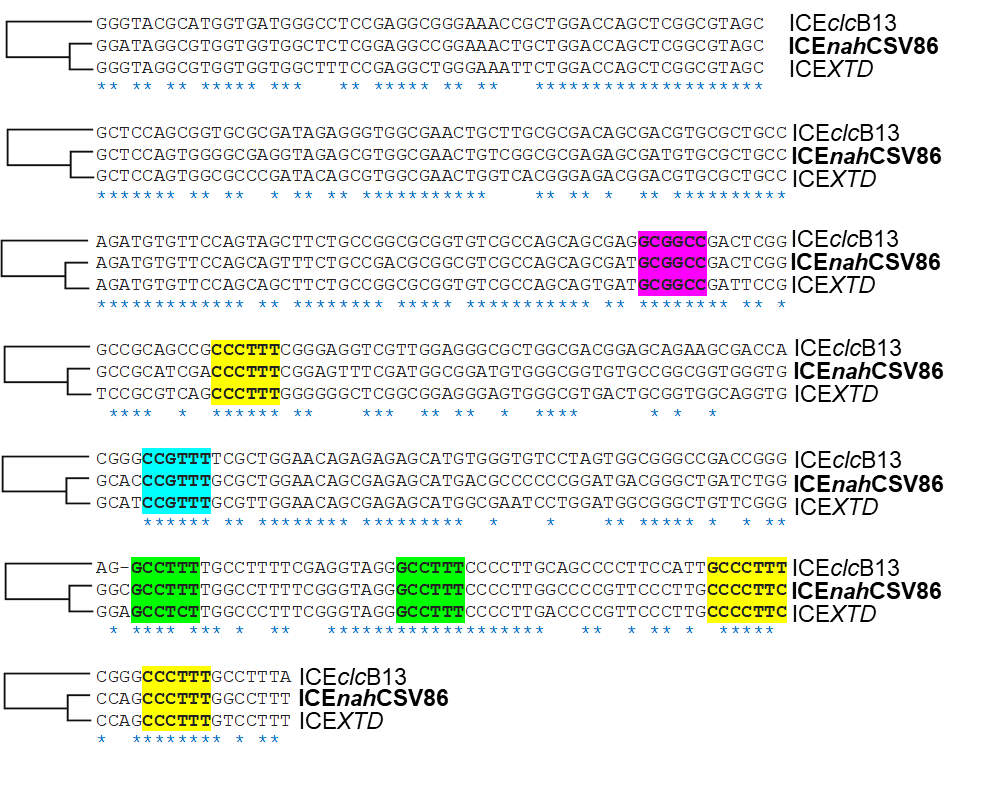
**

**Figure S8.** Prediction of origin of transfer (*ori*T) in ICE*nah*CSV86 and its multiple alignment-based comparison with ICE*clc* members (ICE*clc*B13, ICE*XTD*). The yellow and green highlighted sequences indicate signature 6 bp repeat motifs required for ICE unwinding and transfer, while cyan and purple highlighted 6 bp repeats are accessory motifs. The clade connecting the sequences is a cladogram based on overall distances between the sequences obtained using CLUSTAL-Omega.

**Figure S9**

**A**

**
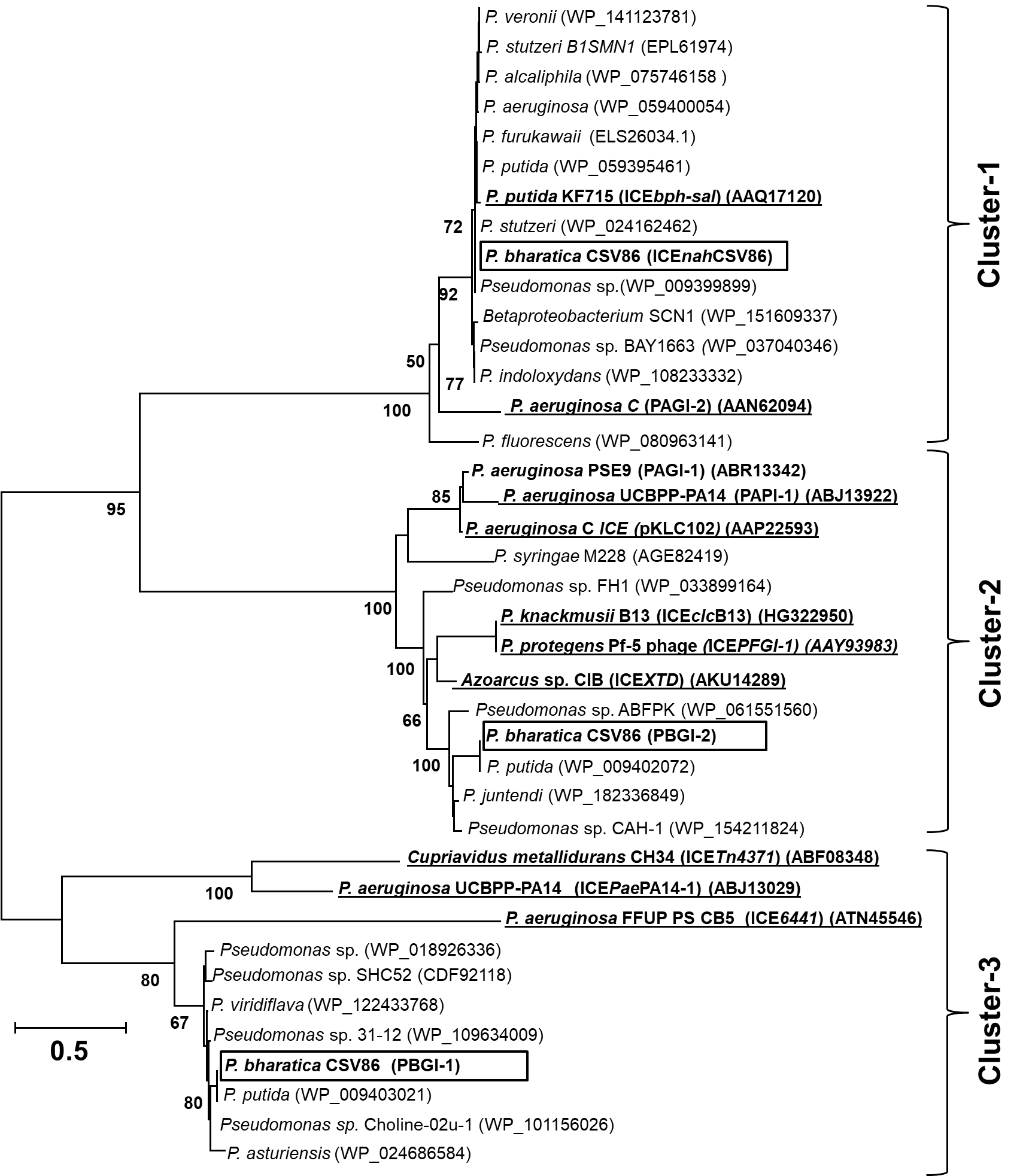
**

**B**

**
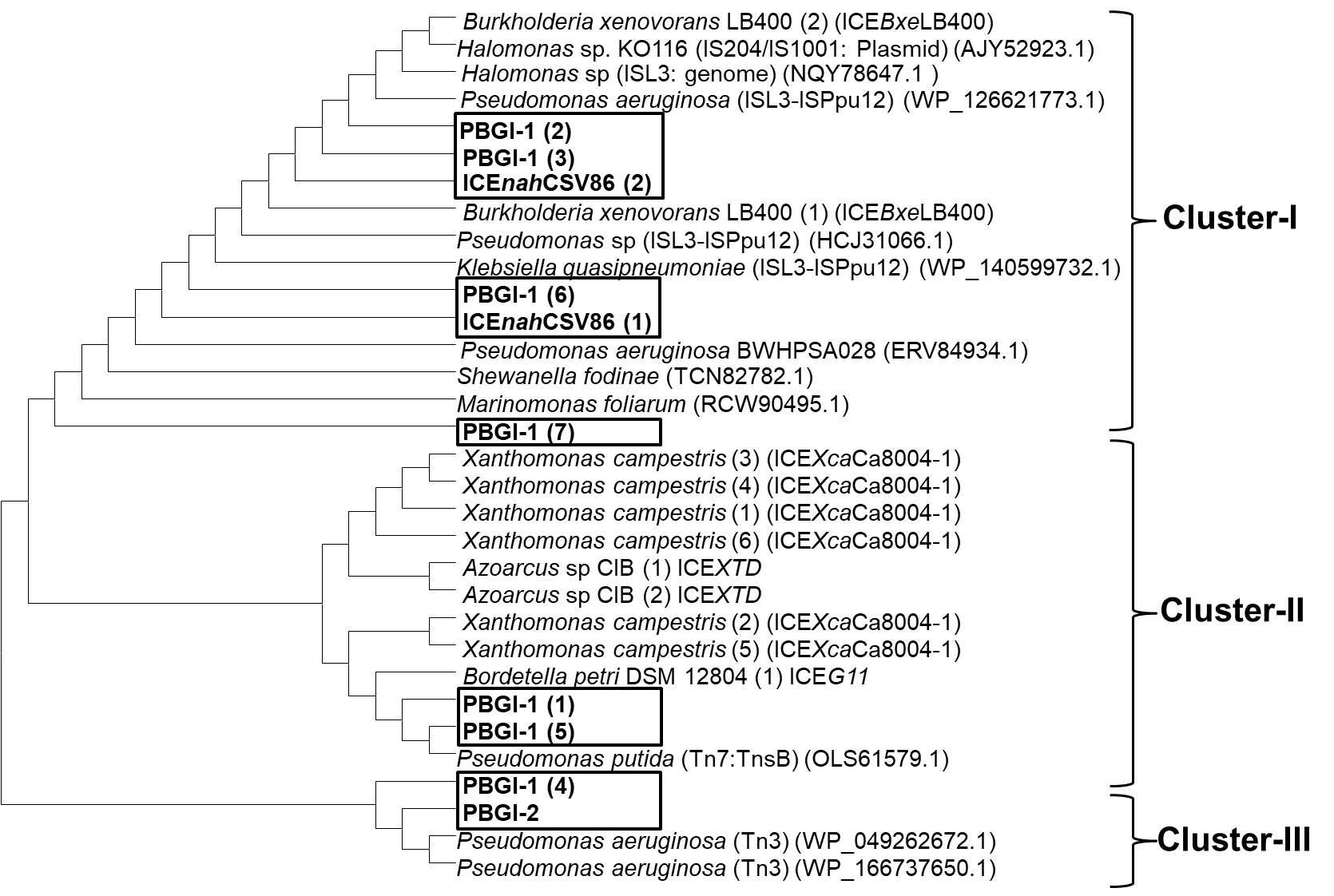
**

**Figure S9.** Phylogenetic reconstruction of core genes from three suspected ICEs of *P. bharatica* CSV86^T^ and other ICEs (characterized: bold and underlined) and non-ICE bearing members. **A)** Maximum-likelihood based phylogeny of integrases, and **B)** *p*-distance based UPGMA cladogram tree of transposases. The suspected ICEs from strain CSV86^T^ are in bold and rectangular boxes. The maximum-likelihood tree was generated involving substitution-based JTT matrix in MEGA 7.0 with 1000 bootstrap iterations and the values at each node are represented as percentage replicates (%). Bar indicates substitution at 0.05 %.

**Figure S10**

**A**

**
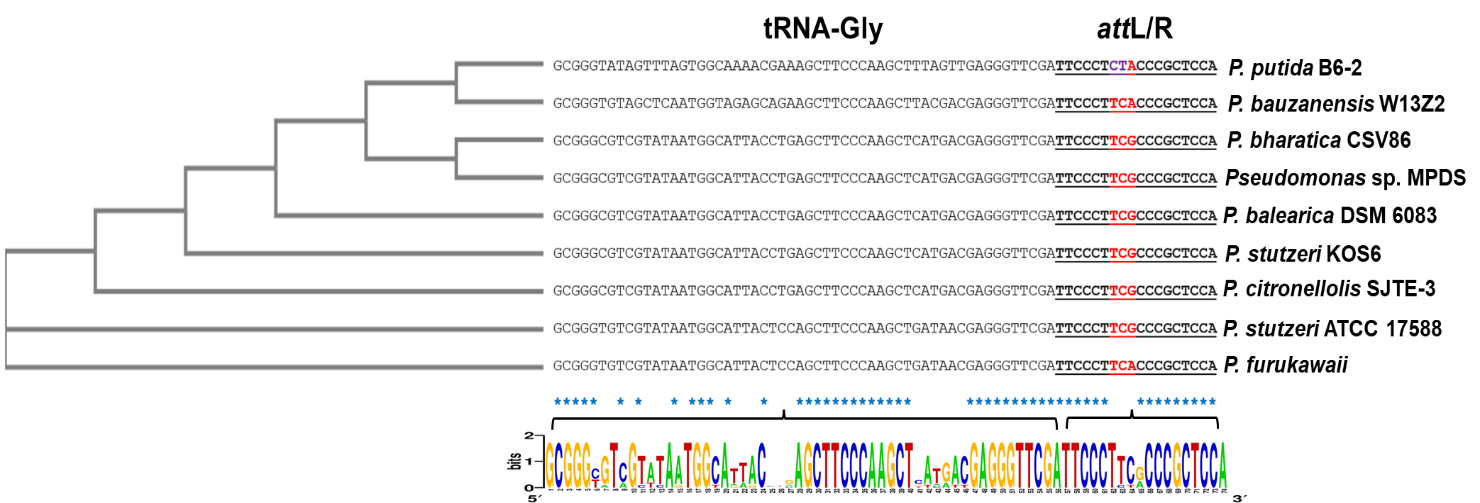
**

**B**

**
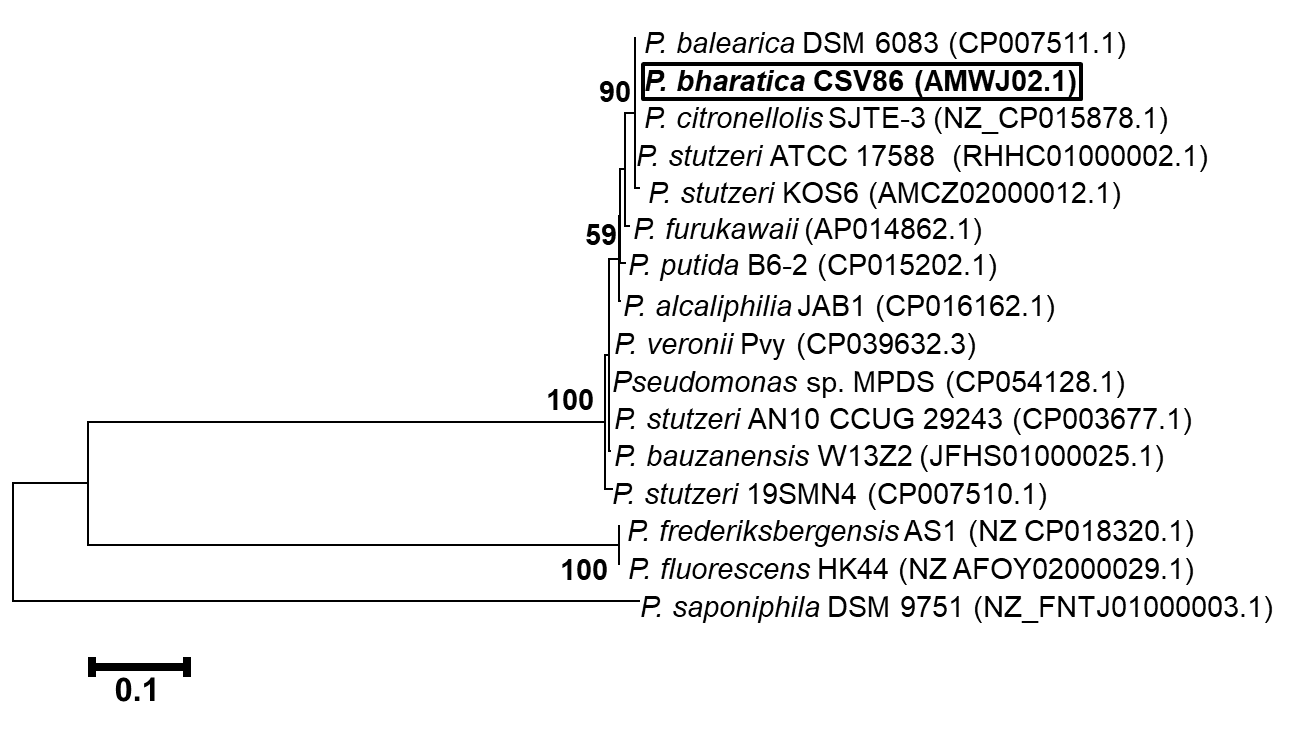
**

**Figure S10.** Alignment and phylogenetic analysis of structural elements of ICE*nah*CSV86 and its comparison with other ICE-bearing (genomically predicted) and non-ICE bearing *Pseudomonas* spp.; **A)** tRNA-Gly and *att*L/R (bold and underlined). The nucleotide residues in black colour indicate highly conserved positions (designated with blue stars below) and those in red colour indicate low to moderately conserved positions (no stars below). **B)** Neighbour-joining phylogenetic tree of integrases (*Int*). Strain CSV86^T^ is depicted in bold and enclosed in rectangular box. Bar indicates substitution at 0.01 %.

**Figure S11**


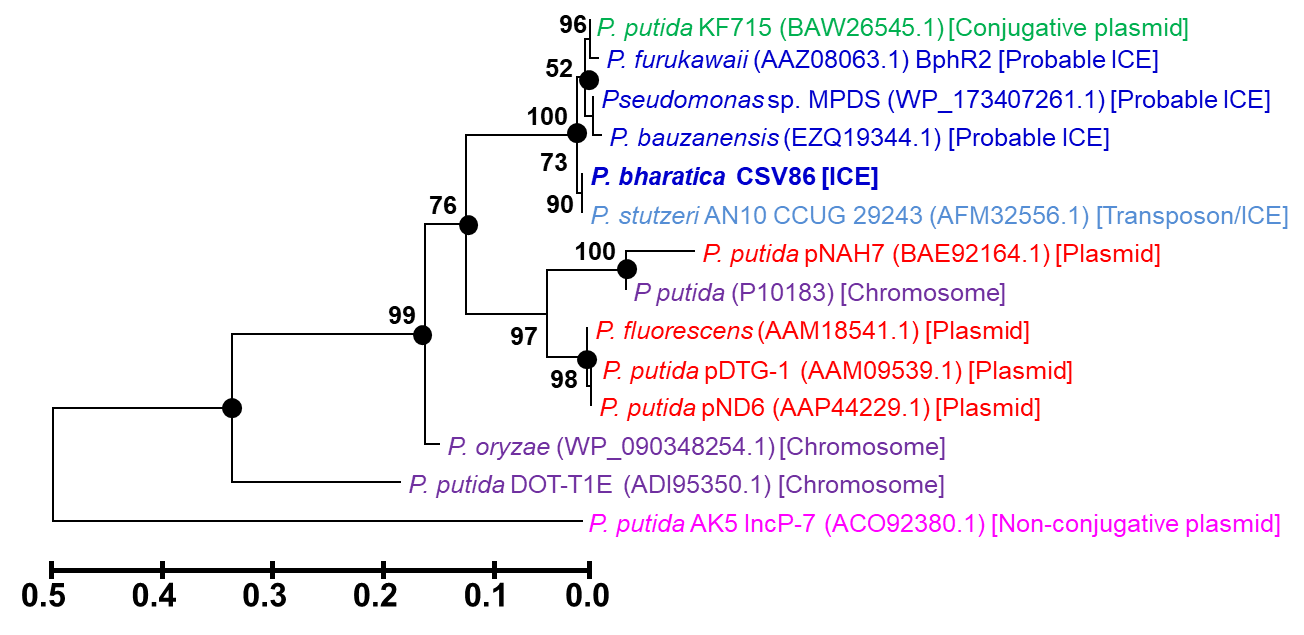


**Figure S11**. Maximum likelihood based phylogenetic reconstruction of NahR. The members bearing NahR are categorized into naphthalene degraders having “*nah*” cluster on different mobile genetic elements: plasmid (red), conjugative plasmid (green), non-conjugative plasmid (purple), transposon (light blue), probable ICE (bright blue), and chromosome (violet). The tree of NahR is represented as optimum tree calculated from maximum likelihood distances at scale of 0-0.05 (% substitution rate) as dendrogram. The black solid circles represent generation of trees from all three tree making algorithms (NJ, ML, ME).

**Figure S12**

**
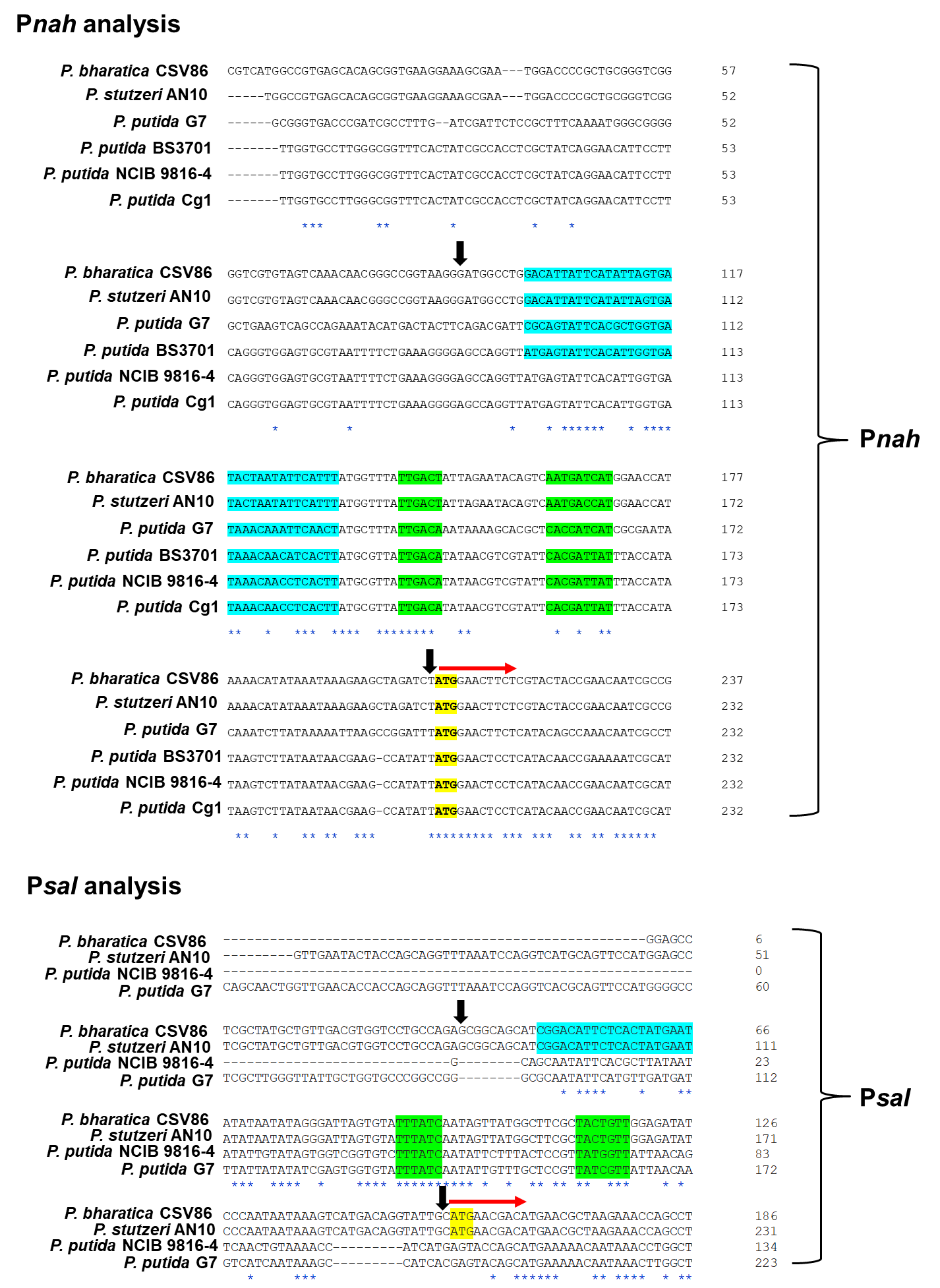
**

**Figure S12.** Multiple sequence alignment of putative strong promoters (designated “P*nah*” and “P*sal*”) present upstream of *nahAa* and *nah*G from various naphthalene degrading organisms. The putative NahR binding sites (-10 and -35 boxes) of *nah*Aa and *nah*G are depicted in turquoise and bright green, respectively. The start codon has been depicted in yellow and red arrow, while the black arrows indicate the regions which are further used for cloning.

**C. Supplementary Tables**

**Table S1.** Primers used to amplify the gap regions of three GIs, suspected to be ICEs, identified after aligning the first draft genome (AMWJ01) sequence with the advanced draft genome (AMWJ02) of *Pseudomonas bharatica* CSV86^T^.

| **GIs** | **Gap regions** | **Primer pairs** | **Sequence (5' to 3')** | **T_m_**  **(°C)** | **Product size (bp)** |
| --- | --- | --- | --- | --- | --- |
| **ICE*nah*CSV86** | Region-1 (C_97 to C_200) | Primer_F1 | TCGCCAGGGGAAGTTTCTATTTGACA | 72.4 | 1618 |
|  |  | Primer_R1 | GATGATTGCCGGTTACGCCACC | 73.1 |  |
|  | Region-2 (C_200 to C_118) | Primer_F2 | GCAGGAGAAACATAGCCGCACG | 71.7 | 460 |
|  |  | Primer_R2 | TCGCTGGGTTGACCATCATTGA | 71.8 |  |
|  | Region-3 (C_118 to C_63) | Primer_F3 | GCGGTCCGGTGACAGCACGTA | 74.7 | 885 |
|  |  | Primer_R3 | AGATTCAGTCTGGCCTCGCGGT | 72.2 |  |
|  | Region-4 (C_63 to C_62) | Primer_F4 | ACCGCGGCACCCACCTCAC | 75.2 | 1576 |
|  |  | Primer_R4 | CAGGTGGGTCAGTTTTACATCGGCCT | 73.7 |  |
|  | Region-5 (C_62 to C_110) | Primer_F5 | TGCGGATCTGCTTCGTGTCCTG | 73.8 | 3523 |
|  |  | Primer_R5 | GACATCCTCACGCTTGTGGACC | 70.2 |  |
| **PBGI-1** | Region-1 (C_129 to C_141) | Primer_F1 | GCAAAGTTGCTGGTGCCATTGG | 72.5 | 400 |
|  |  | Primer_R1 | CATGTGCGGTCGCAGATAACC | 69.7 |  |
|  | Region-2 (C_141 to C_200) | Primer_F2 | TCGGATGGATCTGACTGACACC | 68.6 | 2245 |
|  |  | Primer_R2 | GCGATGATTGCCGGTTACGC | 71.6 |  |
|  | Region-3 (C_200 to C_21) | Primer_F3 | CGACGCTATCACATCACCTTGCG | 72.5 | 586 |
|  |  | Primer_R3 | GGGCCGGTATTGTTGCTTTG | 68.3 |  |
|  | Region-4 (C_21 to C_163) | Primer_F4 | TCGGTGTGAAATGCACTTGGTAGC | 70.8 | 379 |
|  |  | Primer_R4 | GCAATCCCGTCACTCGCACACA | 74.7 |  |
|  | Region-5a (C_163 to C_116) | Primer_F5a | ACGGGACGTCCTGTACACGC | 69.6 | 526 |
|  |  | Primer_R5a | CTGGAGCGATCTGGTCAGGGC | 72.6 |  |
|  | Region-5b (C_116 to C_200) | Primer_F5b | GTTCGCTCCTCAATGTTGGTCAG | 68.9 | 2066 |
|  |  | Primer_R5b | CATGAACCAGAACCGCTTTGACG | 71.5 |  |
|  | Region-5c (C_200 to C_116) | Primer_F5c | CGATGATTGCCGGTTACGCCAC | 74 | 576 |
|  |  | Primer_R5c | CCAGCAGGTCGAGCAGGGTTCG | 76 |  |
|  | Region-5d (C_116 to C_61) | Primer_F5d | CCCGAAGGGCCAAAAGGAAG | 70.6 | 574 |
|  |  | Primer_R5d | CCCTCTGAGCAGCTCCTCGTGG | 73.6 |  |
|  | Region-6 (C_61 to C_64) | Primer_F9 | TCTGGGTGTCATGTCGGGCA | 72.6 | 1426 |
|  |  | Primer_R9 | CGCACAGCATGAAATGGGGCAC | 75.5 |  |
|  | Region-6 (C_64 to C_92) | Primer_F10 | TCGTCTGTGCGCAAGGACAGG | 73.1 | 504 |
|  |  | Primer_R10 | CGACATCCTCCAGTGTGCTGTTA | 68.3 |  |
| **PBGI-2** | Region-1 (C_95 to C_93) | Primer_F1 | CATTTGGGCCGTTCATCTCC | 68.6 | 802 |
|  |  | Primer_R1 | GAGAGTCAGCTGGACCTGGC | 66.6 |  |
|  | Region-2 (C_93 to C_104) | Primer_F2 | CGACGACCTGGTCATCTGG | 67 | 365 |
|  |  | Primer_R2 | GCCATCTGAACCAACCAAGAG | 65.8 |  |
|  | Region-3 (C_104 to C_121) | Primer_F3 | TGCTGGGATGACGATGGTT | 65.7 | 397 |
|  |  | Primer_R3 | CTGAGTAAGGCTCCCTGGCAC | 67.2 |  |

**Table S2.** Primers used to validate: A) polycistronic nature of “*nah*” and “*sal*” operons of ICE*nah*CSV86 through co-transcription analysis and B) to clone P*nah*, P*sal*, P*sal*/*nah*R, and 1NH in pSEVA vector.

| **Name** | **Sequence 5’-3’** |
| --- | --- |
| **A) Co-transcription analysis**  **I*. nah* operon** | |
| *nah*Aa-*nah*Ab-FP | GATTGAACCAACTCGCGGCTATCC |
| *nah*Aa-*nah*Ab-RP | CCTGCCTGTGCAAACATCAAATCTAC |
| *nah*Ab-*nah*Ac-FP | CCGCATGAGTGATGGCTTTCTAGAAGGC |
| *nah*Ab-*nah*Ac-RP | GGACGGAATCAGGCTGTCATGAGTCAG |
| *nah*Ac-*nah*Ad-FP | GGAAACGGAATCGCAAAACGCC |
| *nah*Ac-*nah*Ad-RP | CGGCGCTCGGAAGCTGAG |
| *nah*Ad-*nah*B-FP | CTCCAACCTCATTGTTCACCGAGC |
| *nah*Ad-*nah*B-RP | CCACGACGATCTCGAGTGCG |
| *nah*B-*nah*F-FP | CCCCACATTCGCGTCAACG |
| *nah*B-*nah*F-RP | CTCAGCAACCCTCAGCAGAAGGC |
| *nah*F-*nah*C-FP | GGTTCAGTGCACATTAACGGCTCG |
| *nah*F-*nah*C-RP | CCGTTATGATGGATCACGATTCGATG |
| *nah*C-*nah*E-FP | GTTCTATGGTGCAACGCCTTCGGG |
| *nah*C-*nah*E-RP | CTCACGAGTTTGGCGGATGACTTCG |
| *nah*E-*nah*D-FP | GGACTTGAAAAAGCACGCATGGATG |
| *nah*E-*nah*D-RP | GGAGGGGCCTACGTTTCCGATG |
| **II*. sal* operon** | |
| *nah*G-*nah*T-FP | CGAGTTATACGAGTTGCGCGACC |
| *nah*G-*nah*T-RP | CTTACACAGGCCGCACCCG |
| *nah*T-*nah*H-FP | CGTATGAGCTGCAAGCACGTACCG |
| *nah*T-*nah*H-RP | GGAGAATTTGTCAACTTCGGTCCAAGC |
| *nah*H-*nah*I-FP | CGGCAAGACCATCTACTTCTTCGACC |
| *nah*H-*nah*I-RP | CATCGAAGCGCGCCGTGAC |
| *nah*I-*nah*N-FP | CCGGGCAAATCGAGGCTGG |
| *nah*I-*nah*N-RP | CGATCACCCGACGCGTCTGC |
| *nah*N-*nah*L-FP | GCGTCGACGATCTGGCCAGC |
| *nah*N-*nah*L-RP | GTGCACGCCGAGCATGTTCTG |
| *nah*L-*nah*O-FP | CCAACACCCTCGGCCACTTCG |
| *nah*L-*nah*O-RP | CGCGCTTGAGGCCATCGGAC |
| *nah*O-*nah*M-FP | CAACCGCGTCTCGATCTTCATGG |
| *nah*O-*nah*M-RP | GCTTGAGGCGCGGGATTACC |
| *nah*M-*nah*K-FP | CCAGCGCGCCGAGAAGAAATAC |
| *nah*M-*nah*K-RP | GCGGATTTCCCACTGGATGTCG |
| *nah*K-*nah*J- FP | CGATGCTCGCCAACCTGCTGG |
| *nah*K-*nah*J- RP | CGCCGATGCCGAAGTGGAC |
| *nah*J-*nah*X-FP | CCAGGCCATCTCGCGCTC |
| *nah*J-*nah*X-RP | CATGCGCAGATAGGCCAACGG |
| **B) Cloning and expression** | |
| 1NH-FP | GCTCTAGAAGAGGAGGAAAAATGCTGAAAAATATTTTTTTG (*Xba*I) |
| 1NH-RP | GCGTCGACTTAAAGACAGAGAATTGCCC (*Sal*I) |
| P*nah*-SEVA-FP | TGGTTTAATTAAGATGGCCTGGACATTATTCATATTAGTG (*Pac*I) |
| P*nah*-SEVA-RP | TGGACCTAGGAGATCTAGCTTCTTTATTTATATGTTTTATGG (*Avr*II) |
| P*sal*-SEVA-FP | TGGTTTAATTAAGCGGCAGCATCGGACATTCTCACTATG (*Pac*I) |
| P*sal*-SEVA-RP | TGGAGAATTCGCAATACCTGTCATGACTTTATTATTGGG (*Eco*RI) |
| P*sal*/*nah*R-FP | TGGTTTAATTAATCAATCCGAAAACAGCTCGAACATCAG (*Pac*I) |

Note: The underlined nucleotide sequences indicate recognition sites of the restriction enzymes

**Table S3.** BLASTP (against -nr, and -RefSeq proteins) based homology search showing best homologs of the genes present on ICE*nah*CSV86 of *P. bharatica* CSV86^T^ (presented as spreadsheet data file)

**Table S4.** BLASTP (against -nr, and -RefSeq proteins) based homology search showing best homologs of the genes present on PBGI-1 of *P. bharatica* CSV86^T^ (presented as spreadsheet data file)

**Table S5.** BLASTP (against -nr, and -RefSeq proteins) based homology search showing best homologs of the genes present on PBGI-2 of *P. bharatica* CSV86^T^ (presented as spreadsheet data file)

**Table S6.** Binding and recognition sites for the activator NahR (highlighted in cyan) from various reported naphthalene degrading *Pseudomonas* spp.

| **Organism** | **Binding position from start site (+1)** | **Binding sequence** | **Reference** |
| --- | --- | --- | --- |
| *Pseudomonas putida* plasmid NAH7 | -82 to -47 | CGCAGTATTCACGCTGGTGATAAACAAATTCAACT | Schell & Poser, 1989 |
| *Pseudomonas* sp. | -80 to -47 (RBS) and near -3 (ABS) | TTCA-N_6_-TGAT | Tropel & van der Meer, 2004 |
| Aromatic responsive LTTRs in general | centered at position -66 (RBS) and -27 to -32(ABS) | Characteristic imperfect inverted repeat motif including a T-N_11_-A consensus sequence | Durante-Rodríguez *et al.,* 2017 |
| *Pseudomonas stutzeri* AN10 | -80 to -93 from the translation start site | TTCATATTAGTGA | Bosch *et al.,* 1999 |
| *Pseudomonas putida* plasmid NAH7 | -84 to -30 | CGCAGTATTCACGCTGGTGATAAACAAATTCAACT | Huang & Schell, 1991 |
| Multiple *Pseudomonas* | Not reported | ATTCACGCTN_2_TGAT | Park *et al.,* 2002 |

**Table S7.** Growth attributes demonstrating stability of ICE*nah*CSV86 and PBGI-1 in *P. bharatica* CSV86^T^ in the absence of selection pressure

| **Generations (batch transfers)** | **Benzoate grown inoculum transferred to MSM+Naphthalene** | | | **Benzoate grown inoculum transferred to MSM+Benzoate+Co-Zn-Cd** | |
| --- | --- | --- | --- | --- | --- |
|  | **Specific growth**  **rate (µ, h^-1^)** | **Net biomass yield (mg/L)** | **C23DO sp. activity (nmol/min/mg)** | **Max. growth OD at 24h**  **(1 mM Co-Zn-Cd each)** | **Min. inhibitory Conc. (mM)^*^** |
| 0 (0, control) | 0.36±0.058 | 2120-2142 | 1037±35.65 | 1.56±0.35 | 2.0 |
| 30 (5) | 0.33±0.049 | 1986-2040 | 1016±79.78 | 1.45±0.46 | 2.0 |
| 60 (10) | 0.31±0.053 | 2033-2120 | 1024±57.06 | 1.48±0.39 | 2.0 |

**^*^**The minimum inhibitory concentration (MIC) is determined/tested for each of the individual metals for strain CSV86^T^
